# Supplementary material for: Tunable and low-loss correlated plasmons in Mott-like insulating oxides
Source: Nat Commun. 2017 May 12;8:15271. doi: 10.1038/ncomms15271 (PMC5437299; doi:10.1038/ncomms15271)
Supplement: Supplementary Information — Supplementary Figures, Supplementary Table, Supplementary Notes and Supplementary References [file ncomms15271-s1.pdf]

Supplementary Figures

Supplementary Figure 1

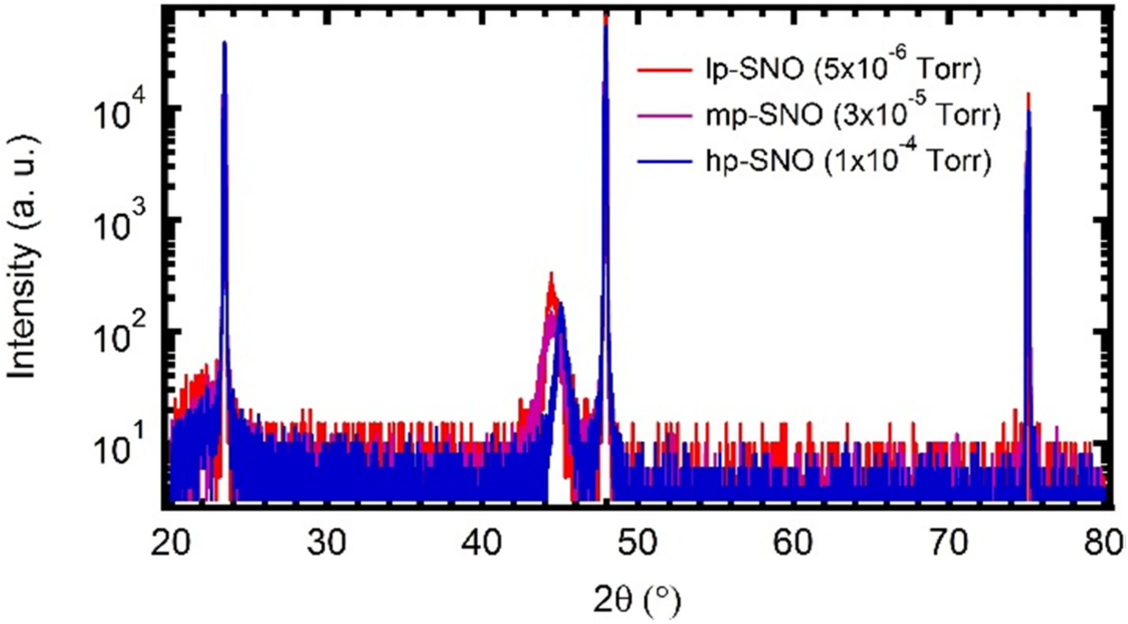

Supplementary Figure 1 | X-ray diffraction spectra of pressure-dependent $Sr_{1-x}NbO_{3+\delta}$ films. The in-plane lattice constant of all films is found to be 4.04 Å. Meanwhile, the out-of-plane lattice constant of lp-SNO and mp-SNO is 4.10 Å whereas for hp-SNO it is 4.02 Å.

Supplementary Figure 2

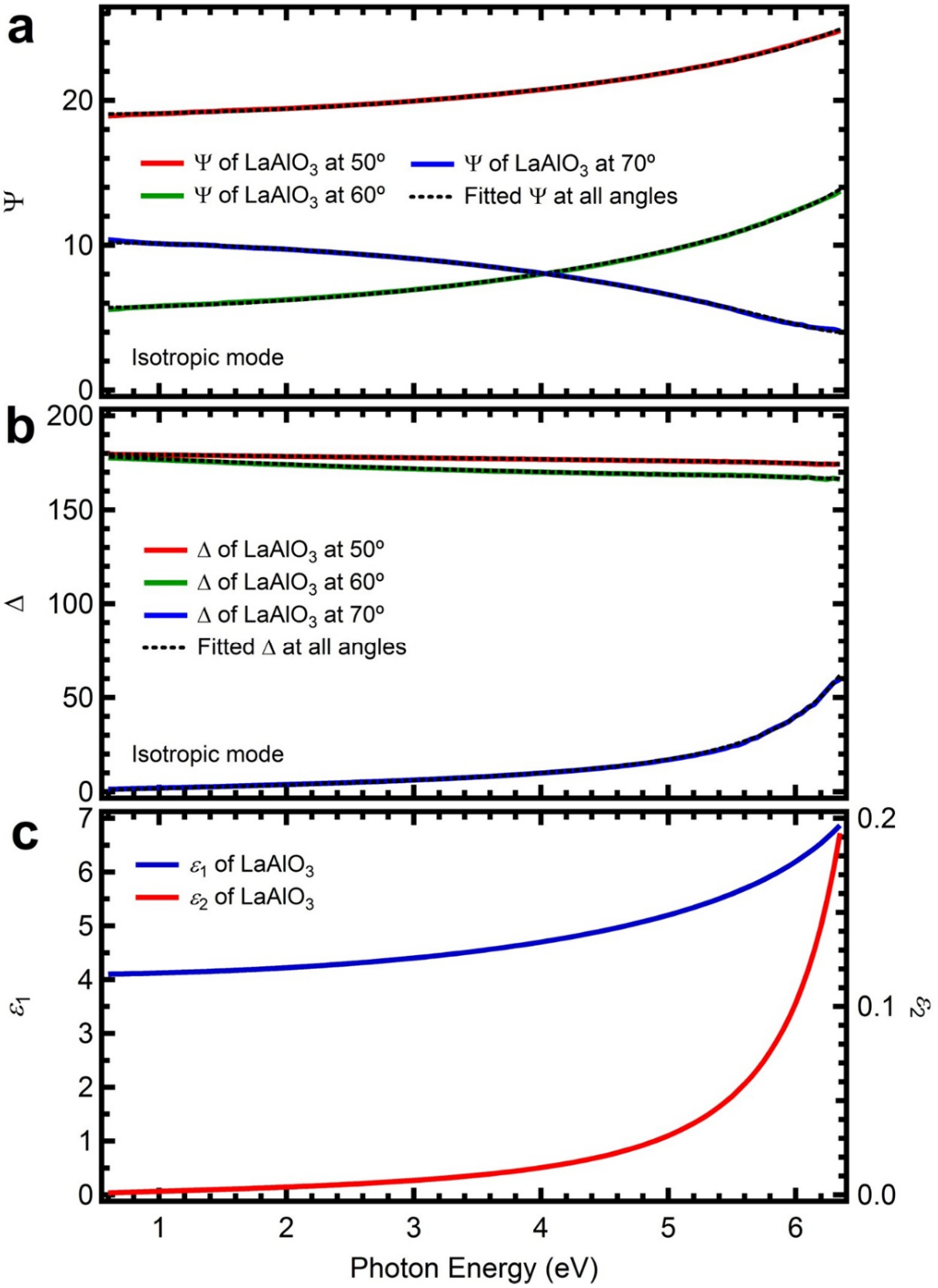

Supplementary Figure 2 | Complex dielectric function analysis of $LaAlO_3$ substrate.

a, Analysis of Ψ of $LaAlO_3$ substrate using isotropic mode. **b**, Analysis of Δ of $LaAlO_3$ substrate using isotropic mode. **c**, Complex dielectric function, $\varepsilon(\omega) = \varepsilon_1(\omega) + i\varepsilon_2(\omega)$, of $LaAlO_3$ substrate extracted from Ψ and Δ.

Supplementary Figure 3

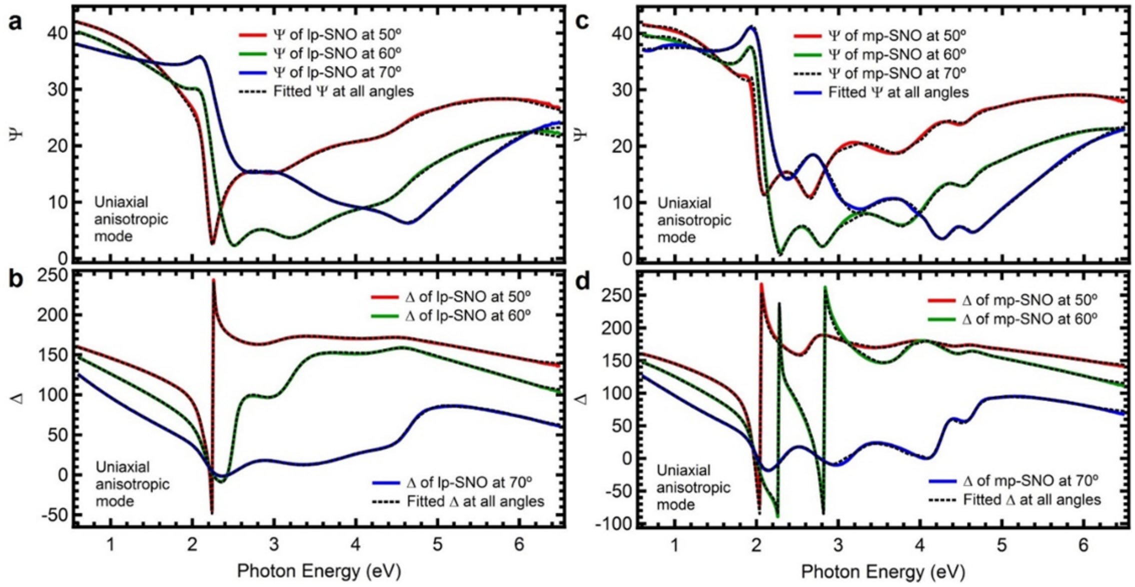

Supplementary Figure 3 | Analyses of spectroscopic ellipsometry data of lp-SNO and mp-SNO. a, Analysis of Ψ of lp-SNO using uniaxial anisotropic mode. **b**, Analysis of Δ of lp-SNO using uniaxial anisotropic mode. **c**, Analysis of Ψ of mp-SNO using uniaxial anisotropic mode. **d**, Analysis of Δ of mp-SNO using uniaxial anisotropic mode.

Supplementary Figure 4

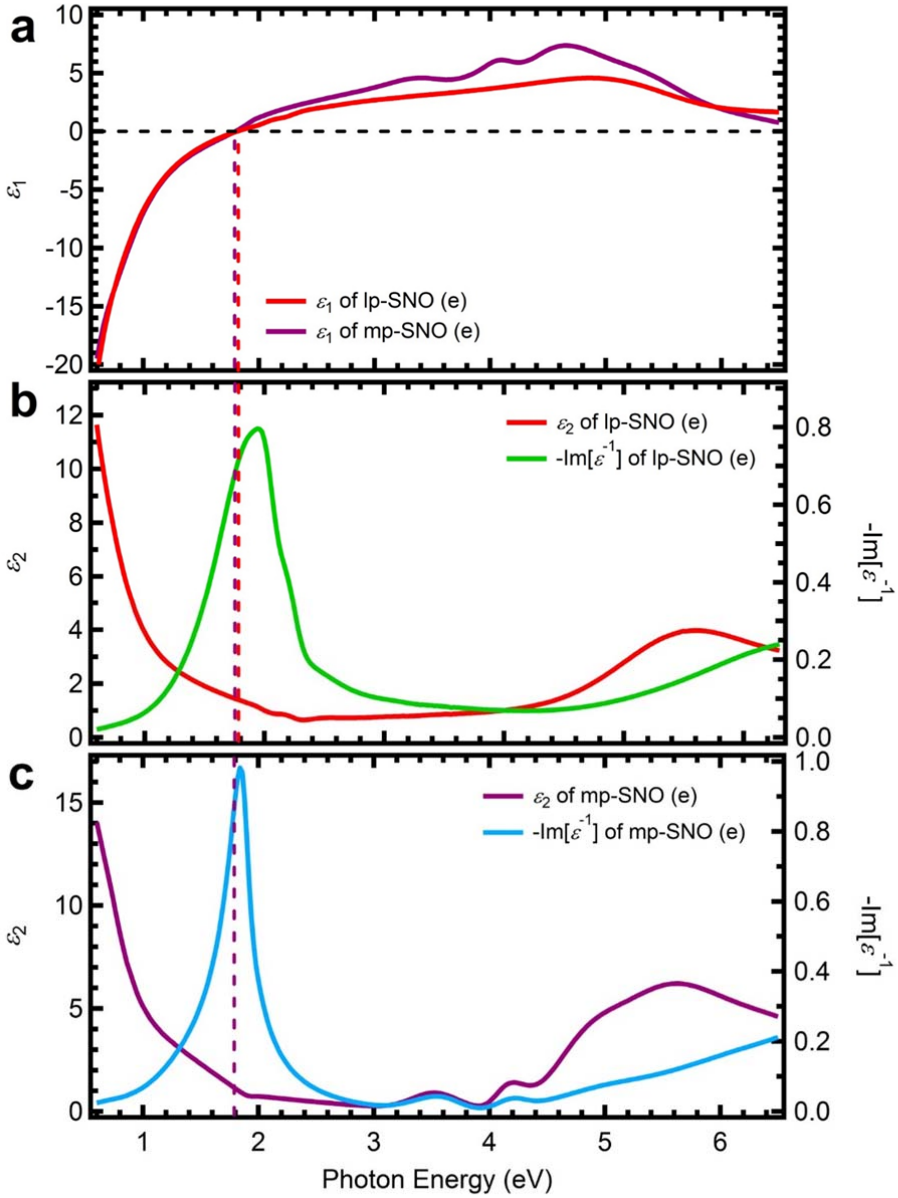

Supplementary Figure 4 | Extraordinary complex dielectric function and loss function spectra of lp-SNO and mp-SNO. **a**, Real part of extraordinary (e) complex dielectric function, $\varepsilon_1(\omega)$, of lp-SNO and mp-SNO. Vertical dashed lines indicate the zero-crossings of extraordinary $\varepsilon_1(\omega)$ of lp-SNO (red) and mp-SNO (purple). **b**, Extraordinary imaginary part of complex dielectric function, $\varepsilon_2(\omega)$, and extraordinary loss function, $-\mathrm{Im}\left[\varepsilon^{-1}(\omega)\right]$, spectra of lp-SNO. **c**, Extraordinary $\varepsilon_2(\omega)$ and $-\mathrm{Im}\left[\varepsilon^{-1}(\omega)\right]$ spectra of mp-SNO.

Supplementary Figure 5

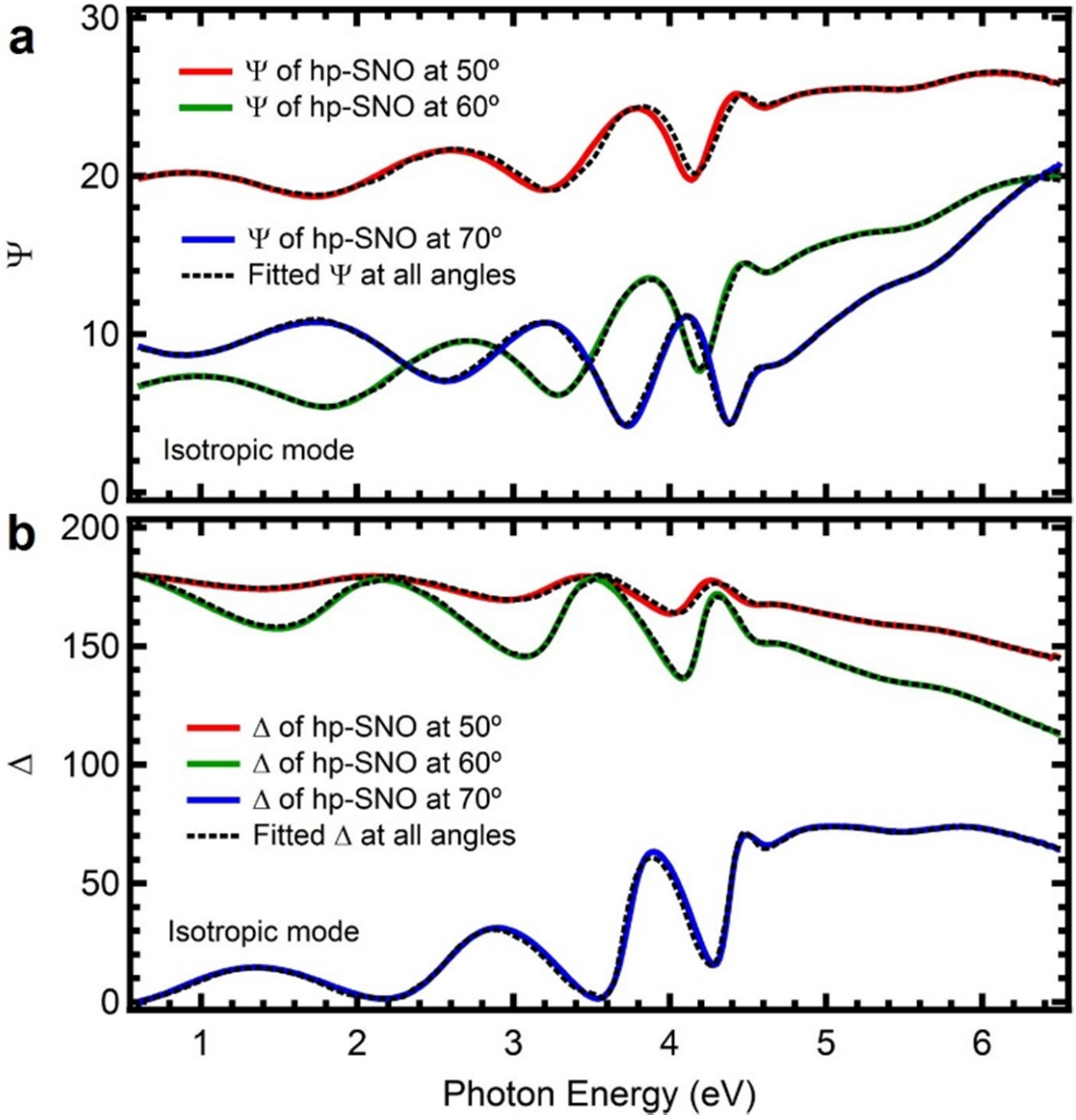

Supplementary Figure 5 | Analyses of spectroscopic ellipsometry data of hp-SNO.

a, Analysis of Ψ of hp-SNO using isotropic mode. **b**, Analysis of Δ of hp-SNO using isotropic mode.

Supplementary Figure 6

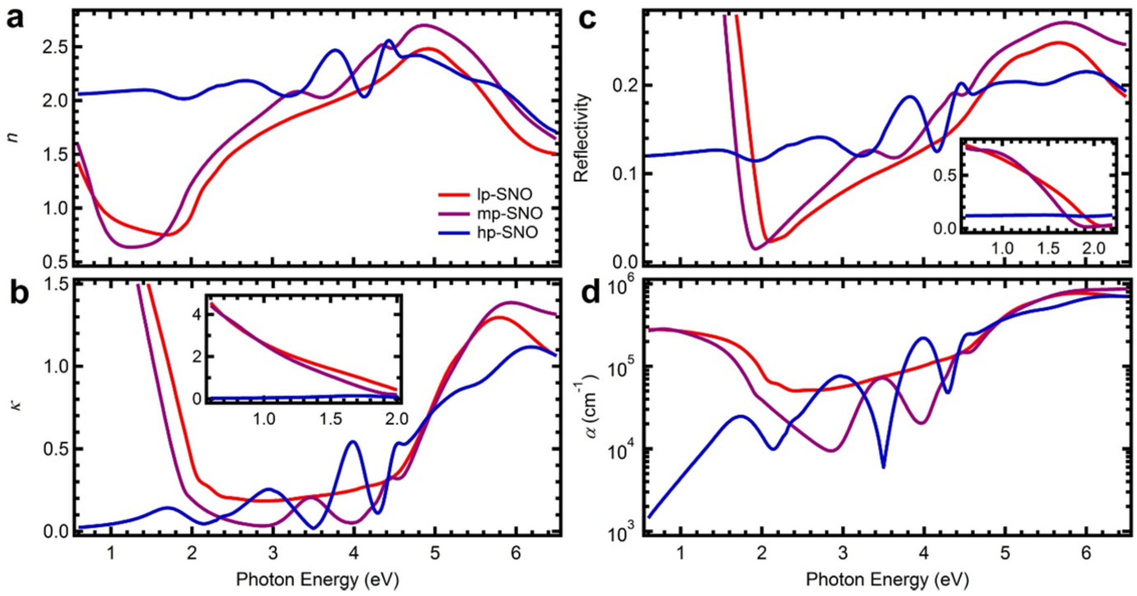

Supplementary Figure 6 | Various optical properties of pressure-dependent $Sr_{1-x}NbO_{3+\delta}$ films. a, Refractive index, *n*, of lp-SNO, mp-SNO, and hp-SNO. **b**, Extinction coefficient, *κ*, of lp-SNO, mp-SNO, and hp-SNO. **c**, Normal-incident reflectivity of lp-SNO, mp-SNO, and hp-SNO. **d**, Absorption coefficient, *α*, of lp-SNO, mp-SNO, and hp-SNO. The legend is shown in **a**. Insets show parts of the spectra zoomed out to show the full extent of the *κ* and normal-incident reflectivity of lp-SNO and mp-SNO.

Supplementary Figure 7

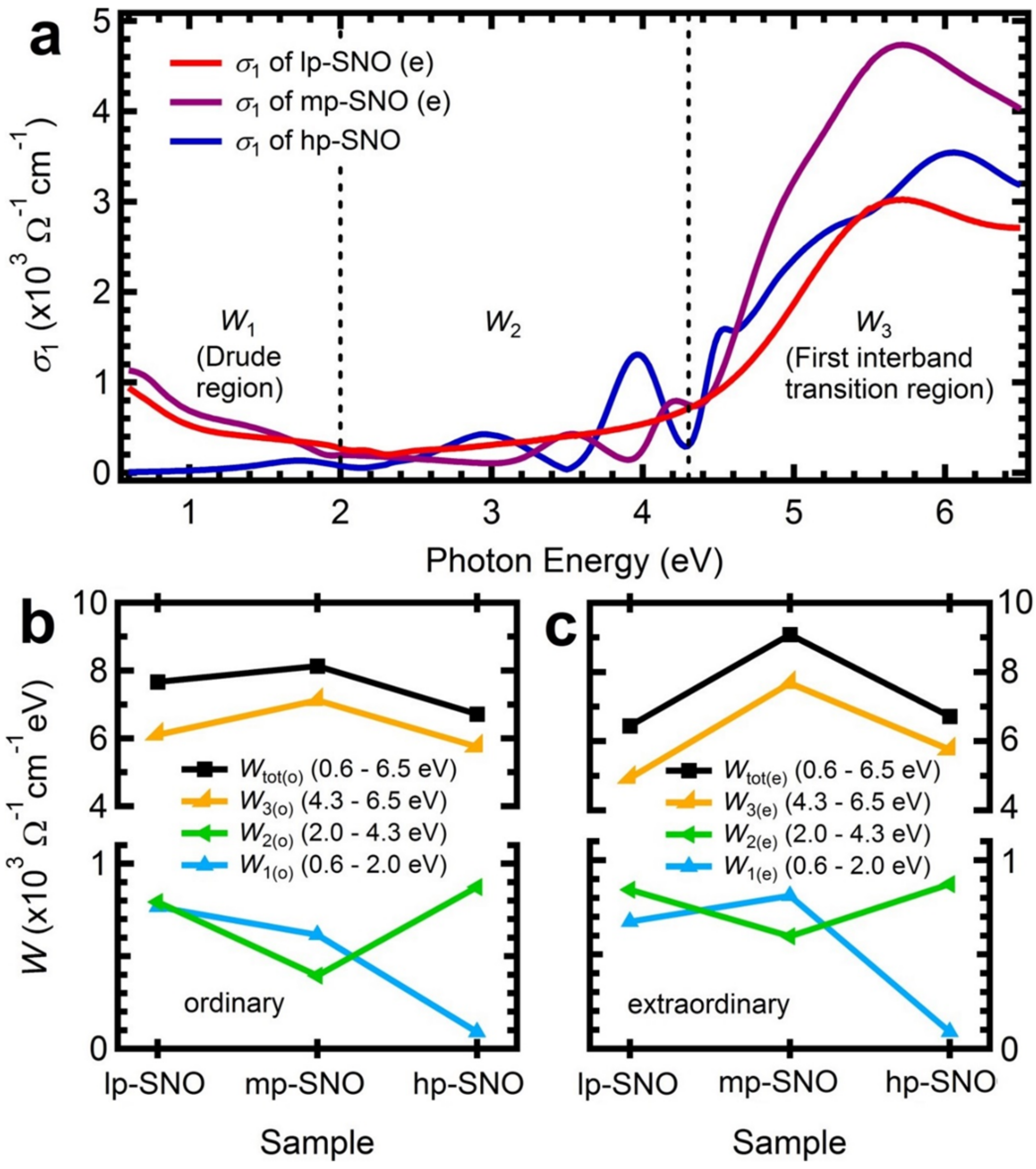

Supplementary Figure 7 | Extraordinary optical conductivity spectra and spectral weight of pressure-dependent $Sr_{1-x}NbO_{3+\delta}$ films. a, The optical conductivity, $\sigma_1(\omega)$, of hp-SNO along with the extraordinary (e) $\sigma_1(\omega)$ of lp-SNO and mp-SNO. **b**, Evolution of ordinary (o) spectral weight, W, of three energy regions: $W_{1(o)}$ (0.6-2.0 eV), $W_{2(o)}$ (2.0-4.3 eV), and $W_{3(o)}$ (4.3-6.5 eV) across the three films. The $W_{tot(o)}$ is the total ordinary W from 0.6 to 6.5 eV. **c,** Evolution of extraordinary W of the three energy regions: $W_{1(e)}$, $W_{2(e)}$, and $W_{3(e)}$, along with the total extraordinary W, $W_{tot(e)}$, across the three films.

Supplementary Figure 8

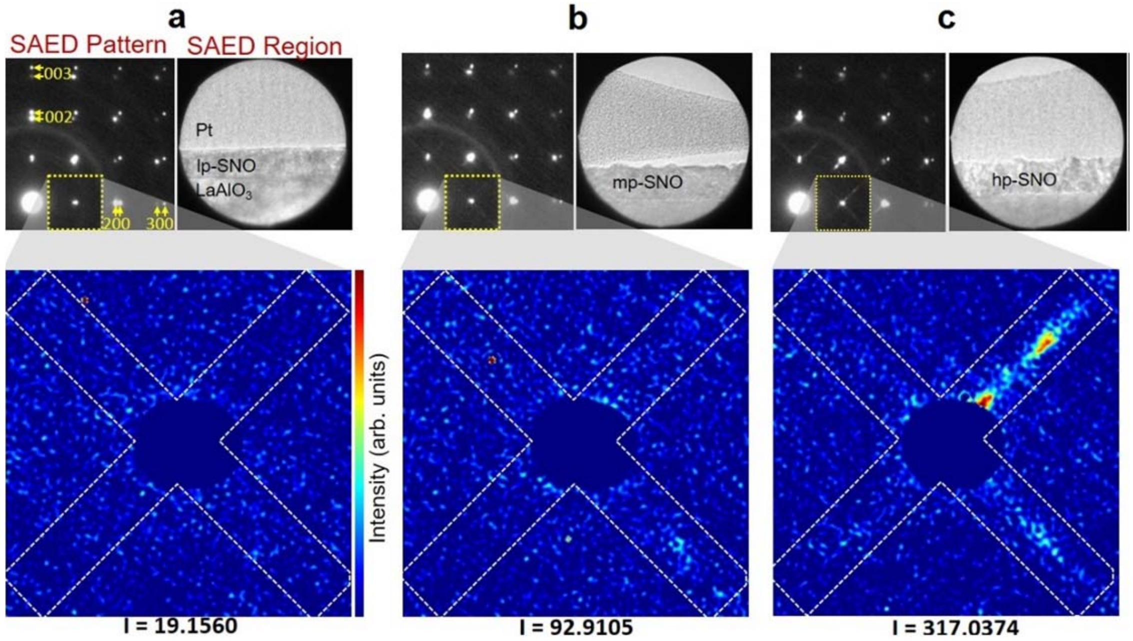

Supplementary Figure 8 | Selected area electron diffraction (SAED) of pressure-dependent $Sr_{1-x}NbO_{3+\delta}$ films. a, The SAED pattern of lp-SNO. **b**, The SAED pattern of mp-SNO. **c**, The SAED pattern of hp-SNO. Depicted for each film is one quadrant of the SAED pattern, a transmission electron microscopy image showing the region used to form the SAED pattern (SAED region) and a magnified view of the (100) peak. The magnified (100) peaks are plotted on identical intensity scales for direct comparison. The intensity of superlattice reflections forming a cross pattern around the primary $SrNbO_3$ Bragg peaks increases with oxygen pressure. None are visible for lp-SNO in **a** and strong reflections appear for hp-SNO in **c**. The mean intensity of the diffraction pattern normalized by the sample thickness within the marked X region around the (100) peaks relative to the region outside, *I*, is 19.2, 92.9, and 317.0 for lp-SNO, mp-SNO, and hp-SNO films, respectively.

Supplementary Figure 9

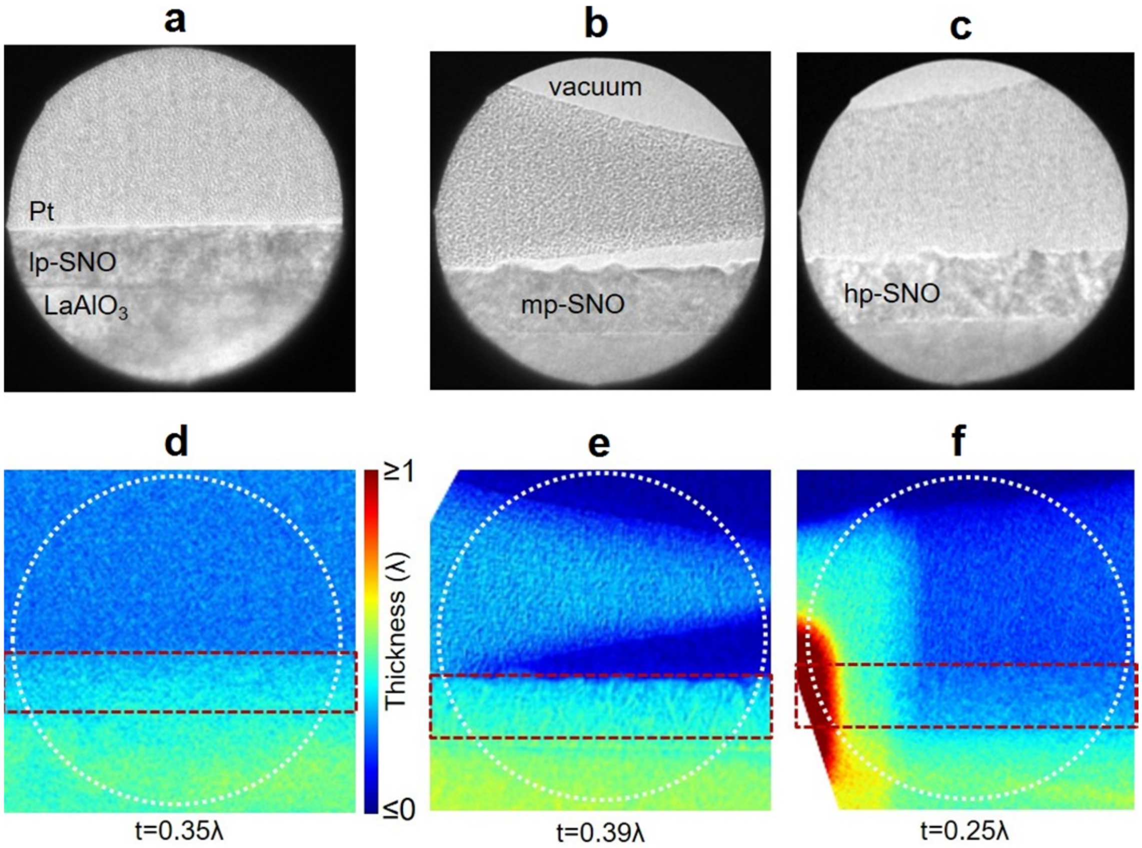

Supplementary Figure 9 | Transmission electron microscopy (TEM) thickness mapping of pressure-dependent $Sr_{1-x}NbO_{3+\delta}$ films. Energy-filtered TEM is used to determine the specimen thickness for the regions used in selected area electron diffraction (SAED) measurements in order to normalize the diffraction volume. **a**, The SAED aperture for lp-SNO. **b**, The SAED aperture for mp-SNO. **c**, The SAED aperture for hp-SNO. **d**, The corresponding thickness map of lp-SNO. **e**, The corresponding thickness map of mp-SNO. **f**, The corresponding thickness map of hp-SNO. The film positions in **d**, **e**, and **f** are highlighted by the dashed red lines. Thickness, t, is measured in units of the mean-electron scattering path, λ. The mean thickness is measured to be ~0.35λ, 0.39λ, and 0.25λ for lp-SNO, mp-SNO, and hp-SNO, respectively. A thick region of carbon contamination is visible on the left edge of **f** which is not present in the corresponding SAED pattern of hp-SNO and ignored in the thickness measurement.

Supplementary Figure 10

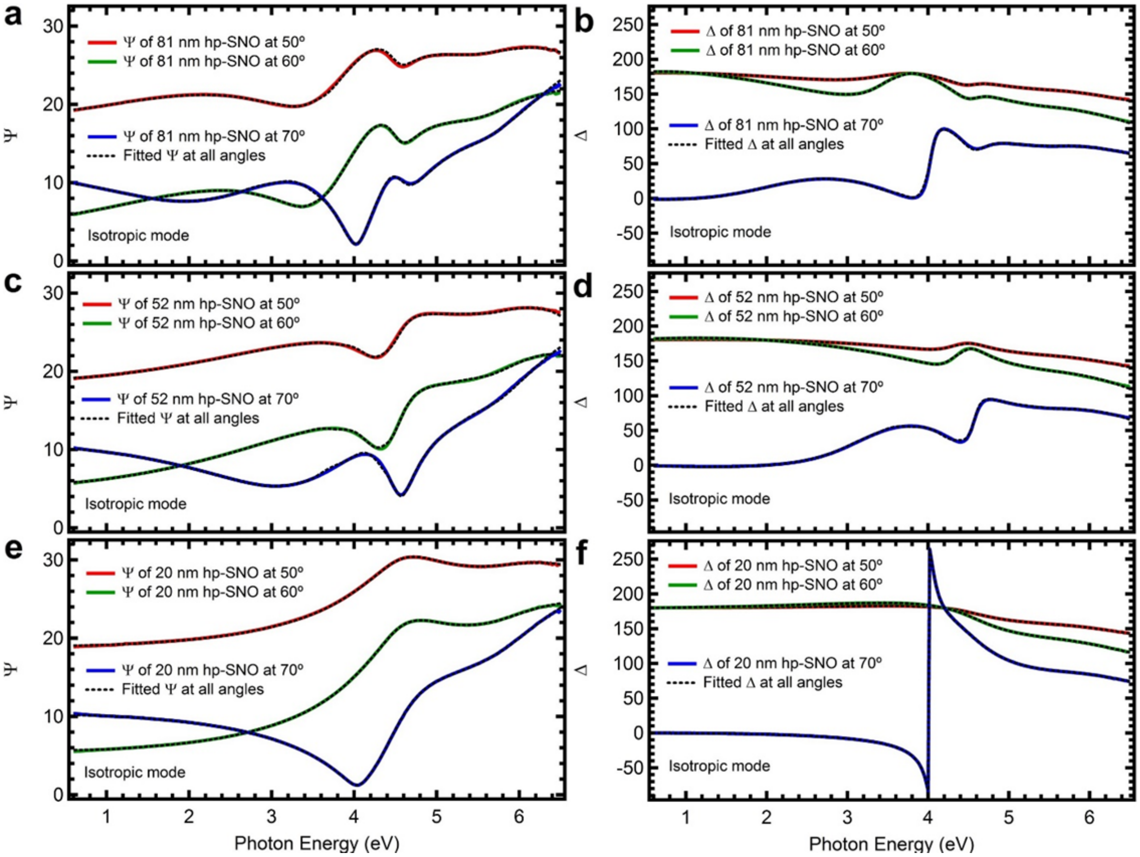

Supplementary Figure 10 | Analyses of spectroscopic ellipsometry data of hp-SNO with varying thicknesses. a, Analysis of Ψ of ~81 nm hp-SNO using isotropic mode. **b**, Analysis of Δ of ~81 nm hp-SNO using isotropic mode. **c**, Analysis of Ψ of ~52 nm hp-SNO using isotropic mode. **d**, Analysis of Δ of ~52 nm hp-SNO using isotropic mode. **e**, Analysis of Ψ of ~20 nm hp-SNO using isotropic mode. **f**, Analysis of Δ of ~20 nm hp-SNO using isotropic mode.

Supplementary Figure 11

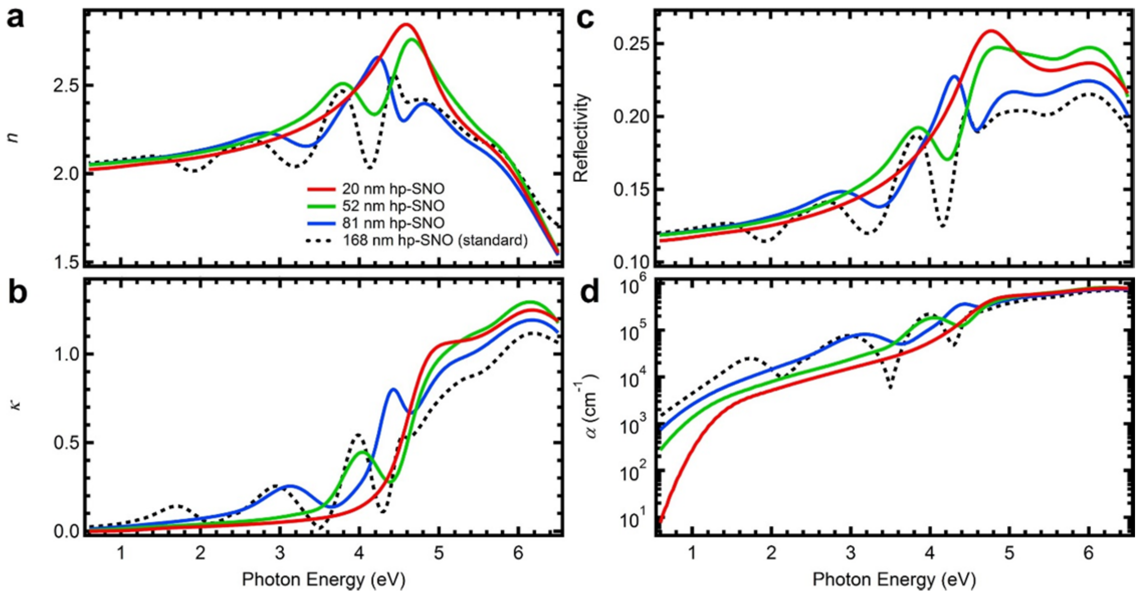

Supplementary Figure 11 | Various optical properties of hp-SNO films with varying thicknesses. a, Refractive index, *n*, of hp-SNO films with varying thicknesses. **b**, Extinction coefficient, *κ*, of hp-SNO films with varying thicknesses. **c**, Normal-incident reflectivity of hp-SNO films with varying thicknesses. **d**, Absorption coefficient, *α*, of hp-SNO films with varying thicknesses. The legend is shown in **a**. The *n*, *κ*, normal-incident reflectivity, and *α* of the standard ~168 nm hp-SNO are also shown for reference.

Supplementary Figure 12

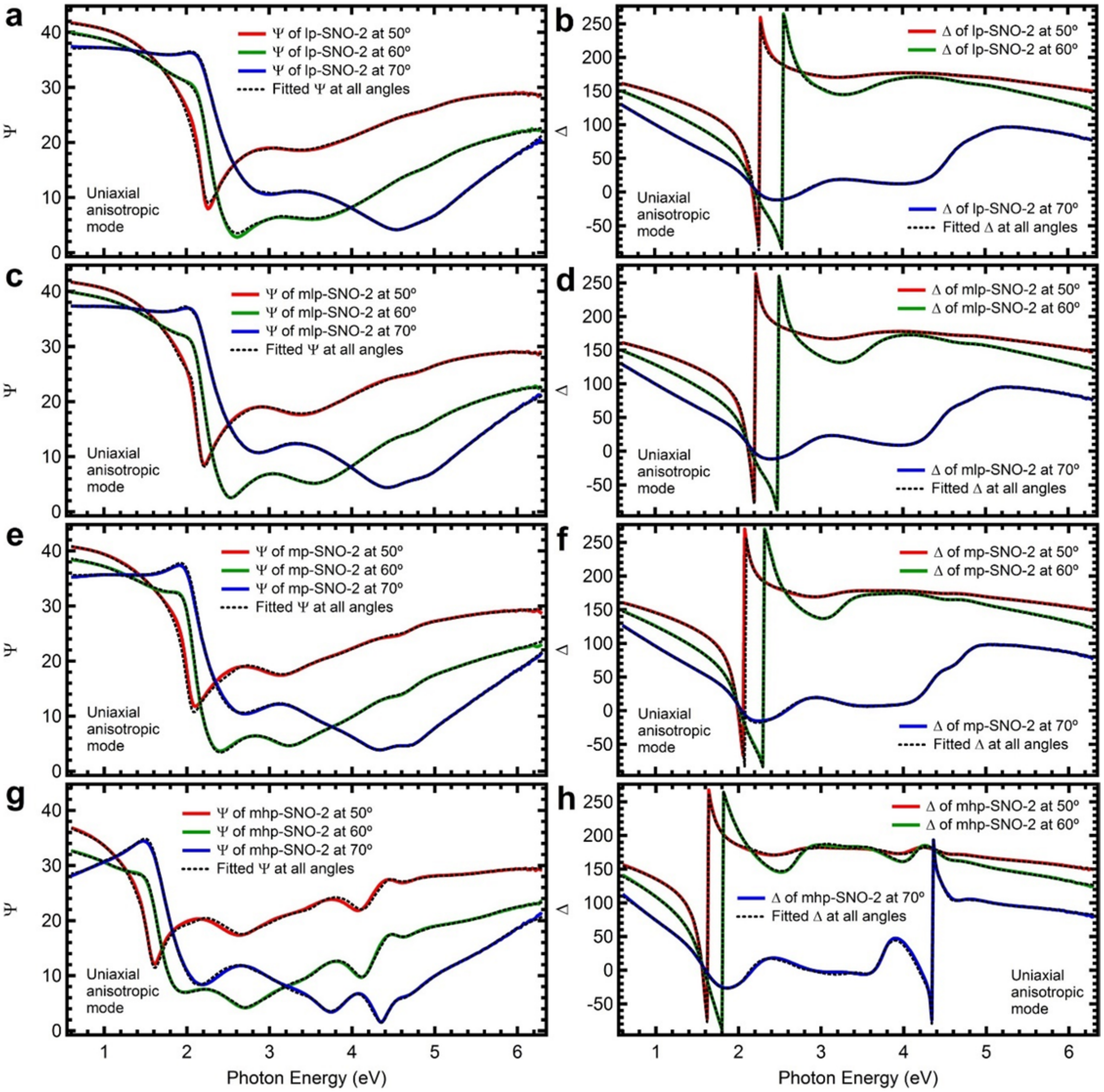

Supplementary Figure 12 | Analyses of spectroscopic ellipsometry data of second-batch pressure-dependent $Sr_{1-x}NbO_{3+\delta}$ films. **a**, Analysis of Ψ of lp-SNO-2 using uniaxial anisotropic mode. **b**, Analysis of Δ of lp-SNO-2 using uniaxial anisotropic mode. **c**, Analysis of Ψ of mlp-SNO-2 using uniaxial anisotropic mode. **d**, Analysis of Δ of mlp-SNO-2 using uniaxial anisotropic mode. **e**, Analysis of Ψ of mp-SNO-2 using uniaxial anisotropic mode. **f**, Analysis of Δ of mp-SNO-2 using uniaxial anisotropic mode. **g**, Analysis of Ψ of mhp-SNO-2 using uniaxial anisotropic mode. **h**, Analysis of Δ of mhp-SNO-2 using uniaxial anisotropic mode.

Supplementary Figure 13

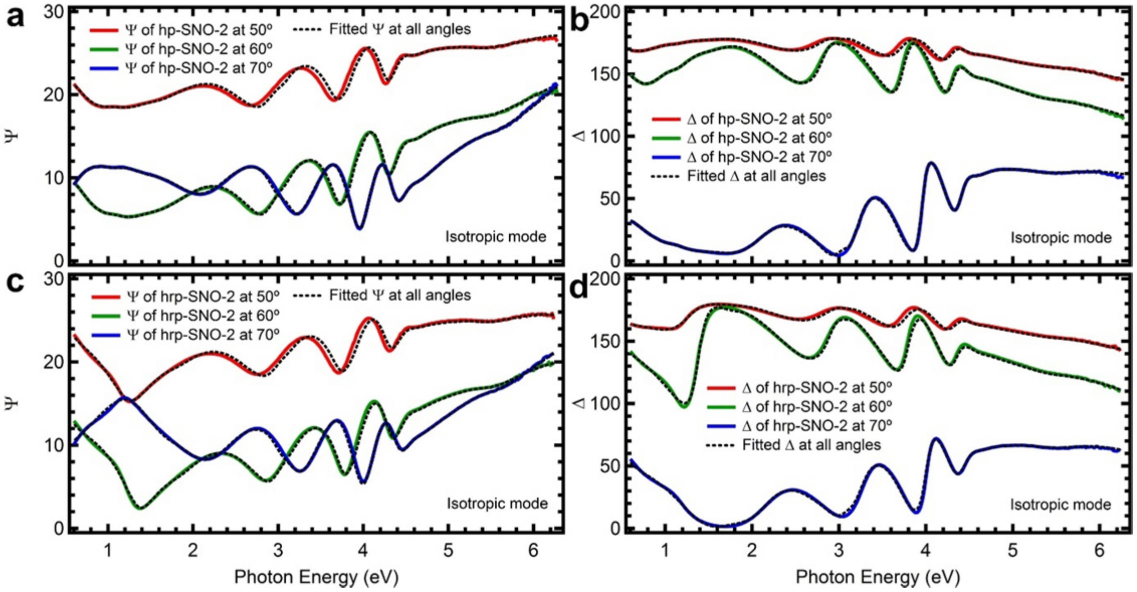

Supplementary Figure 13 | Analyses of spectroscopic ellipsometry data of hp-SNO-2 and hrp-SNO-2. a, Analysis of Ψ of hp-SNO-2 using isotropic mode. **b**, Analysis of Δ of hp-SNO-2 using isotropic mode. **c**, Analysis of Ψ of hrp-SNO-2 using isotropic mode. **d**, Analysis of Δ of hrp-SNO-2 using isotropic mode.

Supplementary Figure 14

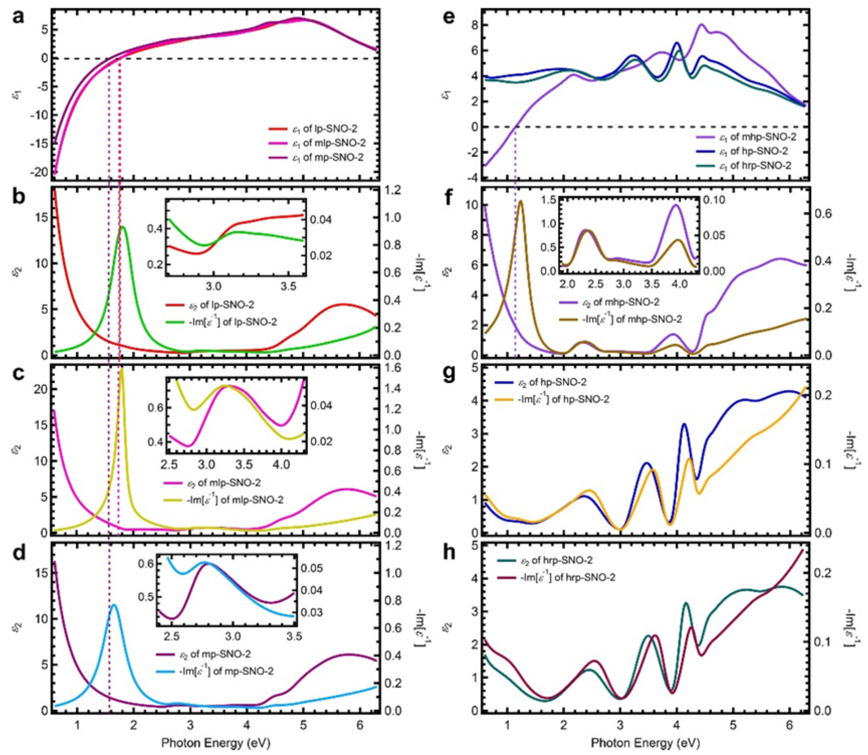

Supplementary Figure 14 | Ordinary complex dielectric function and loss function spectra of second-batch pressure-dependent $Sr_{1-x}NbO_{3+\delta}$ films. a, Real part of ordinary complex dielectric function, $\varepsilon_1(\omega)$, of lp-SNO-2, mlp-SNO-2, and mp-SNO-2. **b**, Imaginary part of ordinary complex dielectric function, $\varepsilon_2(\omega)$, and loss function, $-\mathrm{Im}\left[\varepsilon^{-1}(\omega)\right]$, spectra of lp-SNO-2. **c**, The ordinary $\varepsilon_2(\omega)$ and $-\mathrm{Im}\left[\varepsilon^{-1}(\omega)\right]$ spectra of mlp-SNO-2. **d**, The ordinary $\varepsilon_2(\omega)$ and $-\mathrm{Im}\left[\varepsilon^{-1}(\omega)\right]$ spectra of mp-SNO-2. **e**, The ordinary $\varepsilon_1(\omega)$ spectra of mhp-SNO-2, hp-SNO-2, and hrp-SNO-2. **f**, The ordinary $\varepsilon_2(\omega)$ and $-\mathrm{Im}\left[\varepsilon^{-1}(\omega)\right]$ spectra of mhp-SNO-2. **g**, The ordinary $\varepsilon_2(\omega)$ and $-\mathrm{Im}\left[\varepsilon^{-1}(\omega)\right]$ spectra of hp-SNO-2. **h**, The ordinary $\varepsilon_2(\omega)$ and $-\mathrm{Im}\left[\varepsilon^{-1}(\omega)\right]$ spectra of hrp-SNO-2. Vertical dashed lines indicate the zero-crossings of ordinary $\varepsilon_1(\omega)$ of lp-SNO-2 (red), mlp-SNO-2 (pink), mp-SNO-2 (purple), and mhp-SNO-2 (light violet). Insets show parts of the spectra zoomed in for clarity.

Supplementary Figure 15

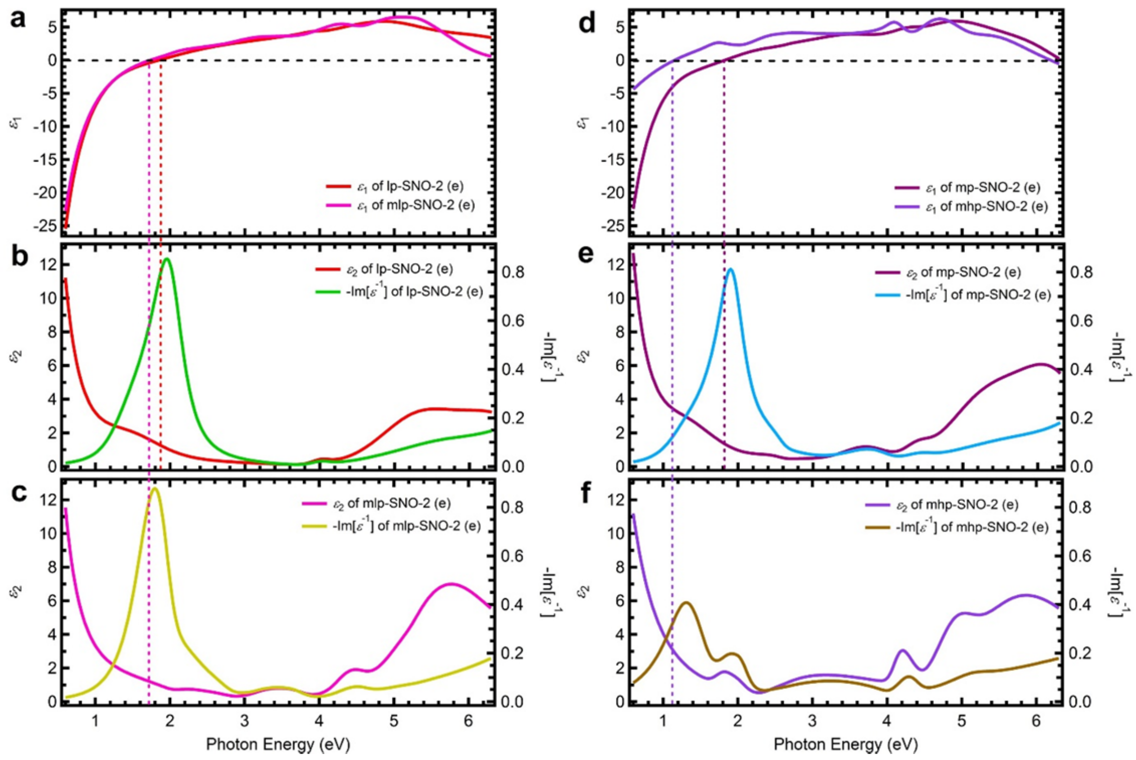

Supplementary Figure 15 | Extraordinary complex dielectric function and loss function spectra of conducting pressure-dependent $Sr_{1-x}NbO_{3+\delta}$ films. a, Real part of extraordinary complex dielectric function, $\varepsilon_1(\omega)$, of lp-SNO-2 and mlp-SNO-2. **b**, Extraordinary imaginary part of complex dielectric function, $\varepsilon_2(\omega)$, and extraordinary loss function, $-\mathrm{Im}\left[\varepsilon^{-1}(\omega)\right]$, spectra of lp-SNO-2. **c**, Extraordinary $\varepsilon_2(\omega)$ and $-\mathrm{Im}\left[\varepsilon^{-1}(\omega)\right]$ spectra of mlp-SNO-2. **d**, Extraordinary $\varepsilon_1(\omega)$ of mp-SNO-2 and mhp-SNO-2. **e**, Extraordinary $\varepsilon_2(\omega)$ and $-\mathrm{Im}\left[\varepsilon^{-1}(\omega)\right]$ spectra of mp-SNO-2. **f**, Extraordinary $\varepsilon_2(\omega)$ and $-\mathrm{Im}\left[\varepsilon^{-1}(\omega)\right]$ spectra of mhp-SNO-2. Vertical dashed lines indicate the zero-crossings of extraordinary $\varepsilon_1(\omega)$ of lp-SNO-2 (red), mlp-SNO-2 (pink), mp-SNO-2 (purple), and mhp-SNO-2 (light violet).

Supplementary Figure 16

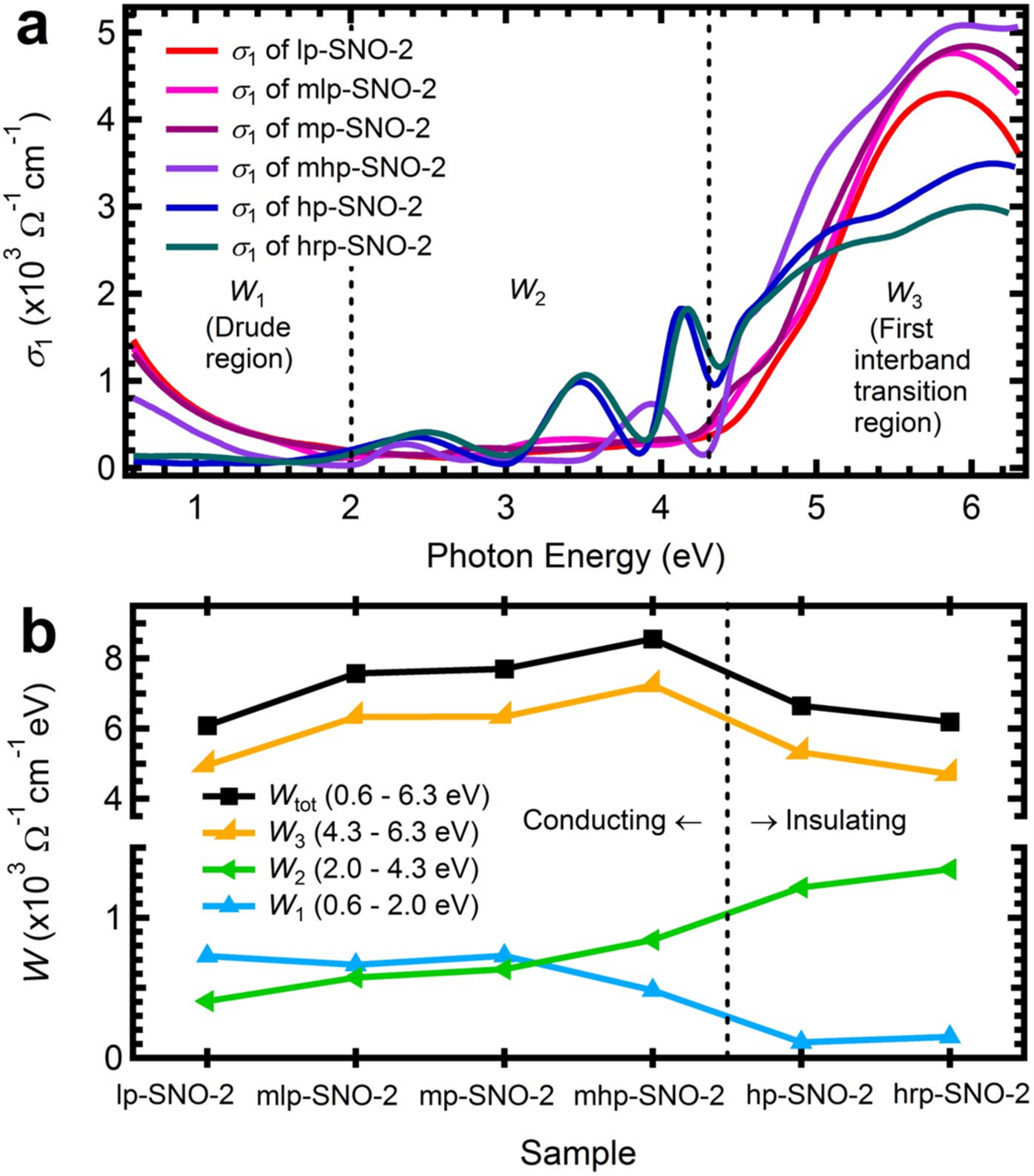

Supplementary Figure 16 | Ordinary optical conductivity spectra and spectral weight of second-batch pressure-dependent $Sr_{1-x}NbO_{3+\delta}$ films. a, The ordinary optical conductivity, $\sigma_1(\omega)$, of lp-SNO-2, mlp-SNO-2, mp-SNO-2, mhp-SNO-2, hp-SNO-2, and hrp-SNO-2. **b**, Evolution of spectral weight, W, of three energy regions: W_1 (0.6-2.0 eV), W_2 (2.0-4.3 eV), and W_3 (4.3-6.3 eV) across the three films. The W_{tot} is the total W from 0.6 to 6.3 eV.

Supplementary Figure 17

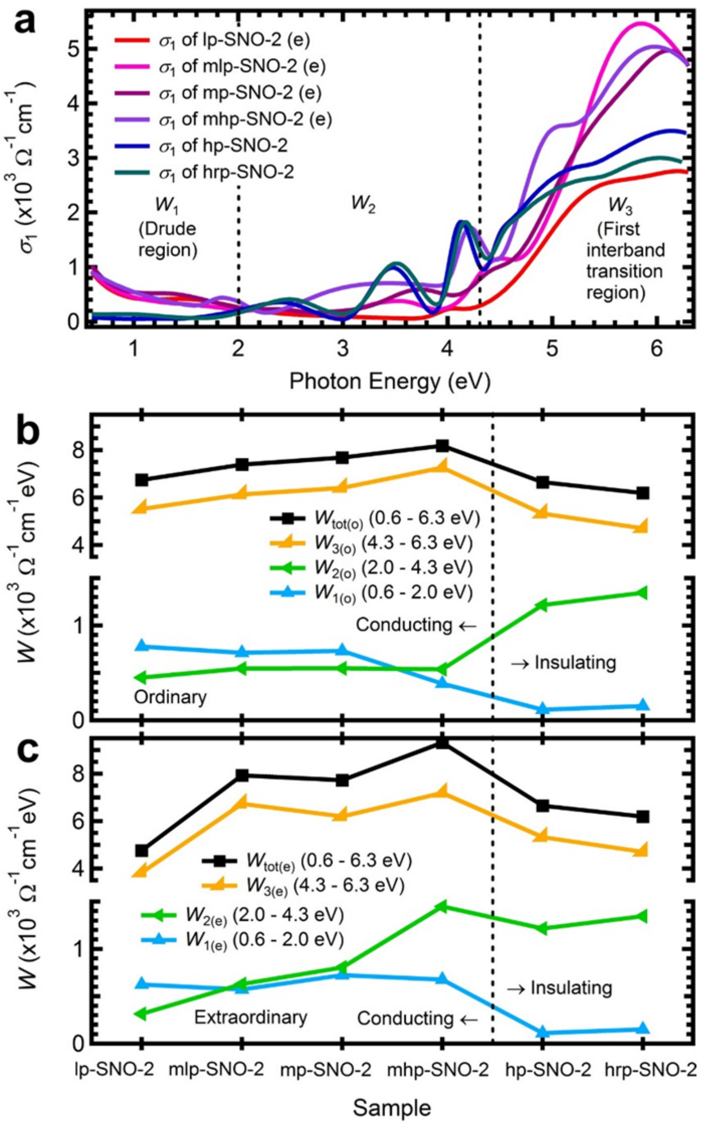

Supplementary Figure 17 | Extraordinary optical conductivity spectra and spectral weight of the second-batch pressure-dependent $Sr_{1-x}NbO_{3+\delta}$ films. a, The optical conductivity, $\sigma_1(\omega)$, of hp-SNO-2 and hrp-SNO-2 along with the extraordinary (e) $\sigma_1(\omega)$ of lp-SNO, mlp-SNO-2, mp-SNO, and mhp-SNO-2. **b**, Evolution of ordinary (o) spectral weight, *W*, of three energy regions: $W_{1(o)}$ (0.6-2.0 eV), $W_{2(o)}$ (2.0-4.3 eV), and $W_{3(o)}$ (4.3-6.3 eV) across the second-batch films. The $W_{tot(o)}$ is the total ordinary *W* from 0.6 to 6.3 eV. **c,** Evolution of extraordinary *W* of the three energy regions: $W_{1(e)}$, $W_{2(e)}$, and $W_{3(e)}$, along with the total extraordinary *W*, $W_{tot(e)}$, across the second-batch films.

Supplementary Figure 18

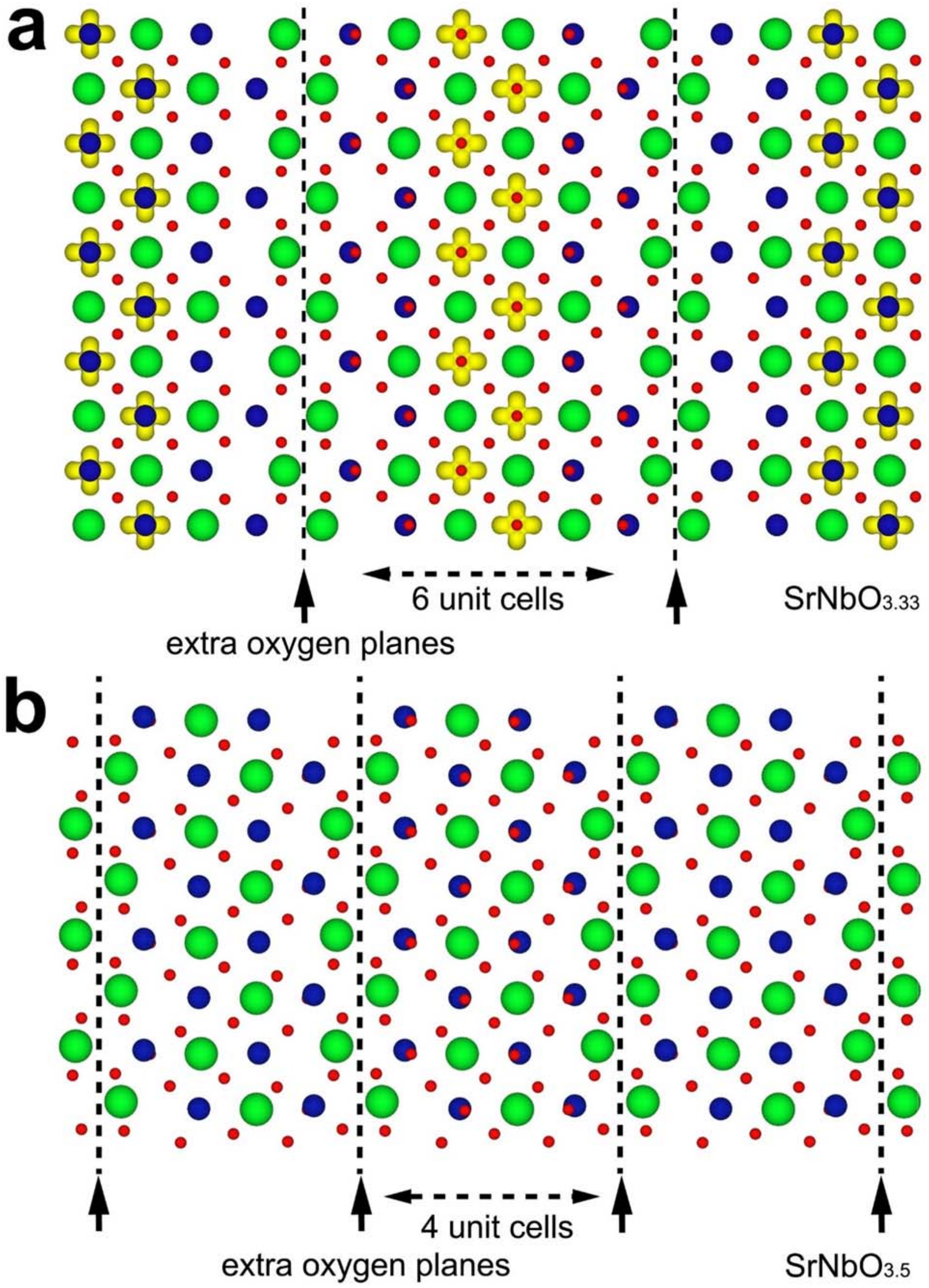

Supplementary Figure 18 | Theoretical Nb-4*d* electron density iso-surface of $SrNbO_{3.33}$ and $SrNbO_{3.5}$. a, Theoretical Nb-4*d* electron density iso-surface of $SrNbO_{3.33}$ superimposed on $SrNbO_{3.33}$ crystal structure (Sr = green, Nb = blue, O = red, iso-surface = yellow). **b**, Theoretical Nb-4*d* electron density iso-surface of $SrNbO_{3.5}$ superimposed on $SrNbO_{3.5}$ crystal structure. Black dashed lines denote the oxygen walls.

Supplementary Figure 19

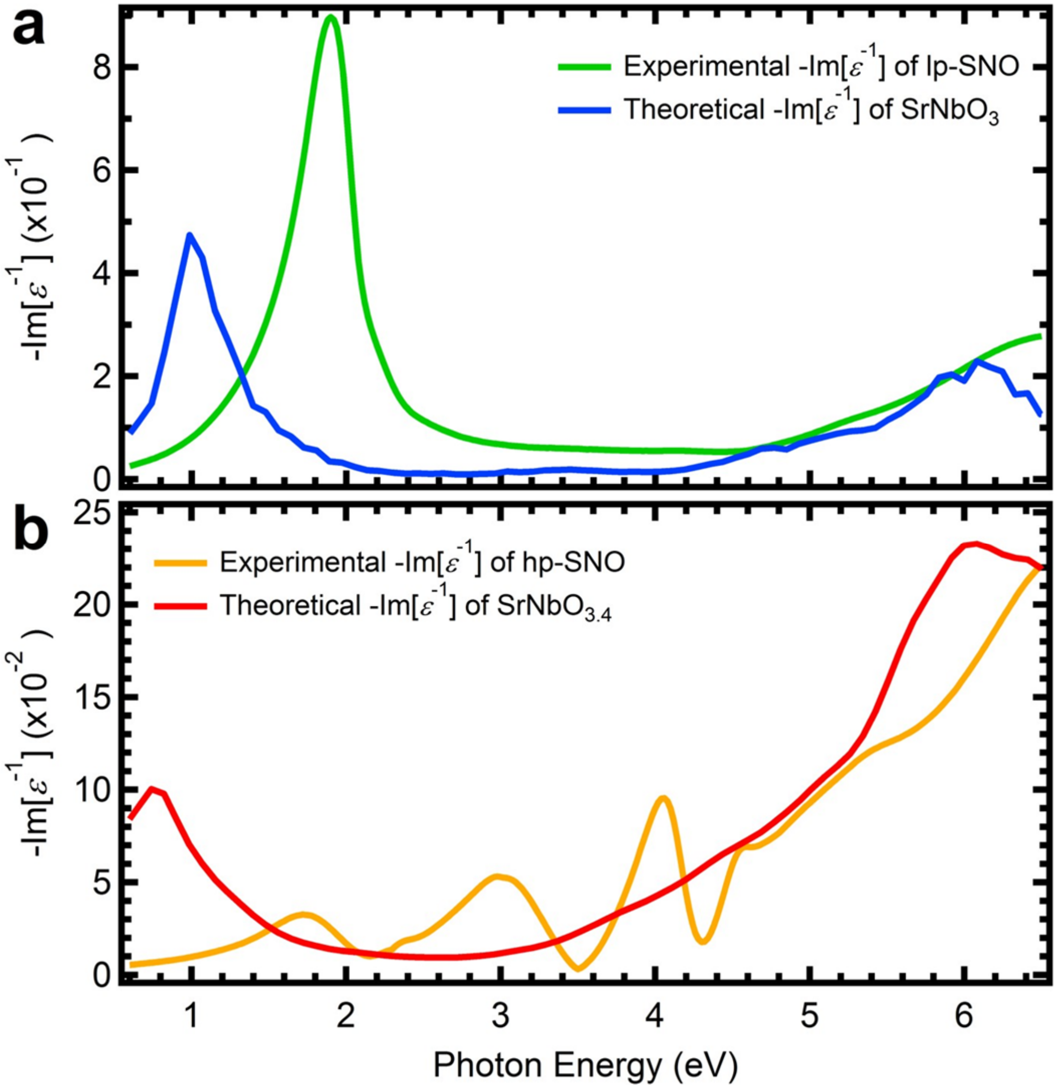

Supplementary Figure 19 | Experimental loss function (LF) of lp-SNO and hp-SNO films compared with theoretical LF of $SrNbO_3$ and $SrNbO_{3.4}$. a, Experimental LF, $-\mathrm{Im}\left[\varepsilon^{-1}(\omega)\right]$, of lp-SNO compared with theoretical LF of $SrNbO_3$. **b**, Experimental LF, $-\mathrm{Im}\left[\varepsilon^{-1}(\omega)\right]$, of hp-SNO compared with theoretical LF of $SrNbO_{3.4}$. The theoretical LF spectra are calculated using density functional theory (DFT) with random phase approximation (RPA) without including strong correlation effects. We note that in RPA, plasmons are treated as superpositions of electron-hole pairs of bare electrons without spectral weight transfer.

Supplementary Figure 20

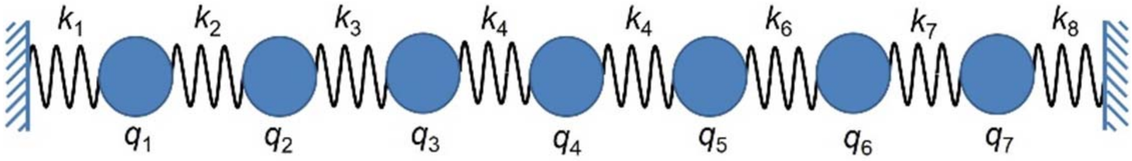

Supplementary Figure 20 | Classical analog of the coupled harmonic oscillator model of strongly-correlated plasmons. Each q_i represents a renormalized quasi-electron with an effective mass and charge that might be different from those of bare electron, while each k_i represents the spring-like Coulomb interaction that couples the quasi-electrons. The extra oxygen planes that confine the quasi-electrons are represented by the immobile walls at the ends of the quasi-electron chain.

Supplementary Figure 21

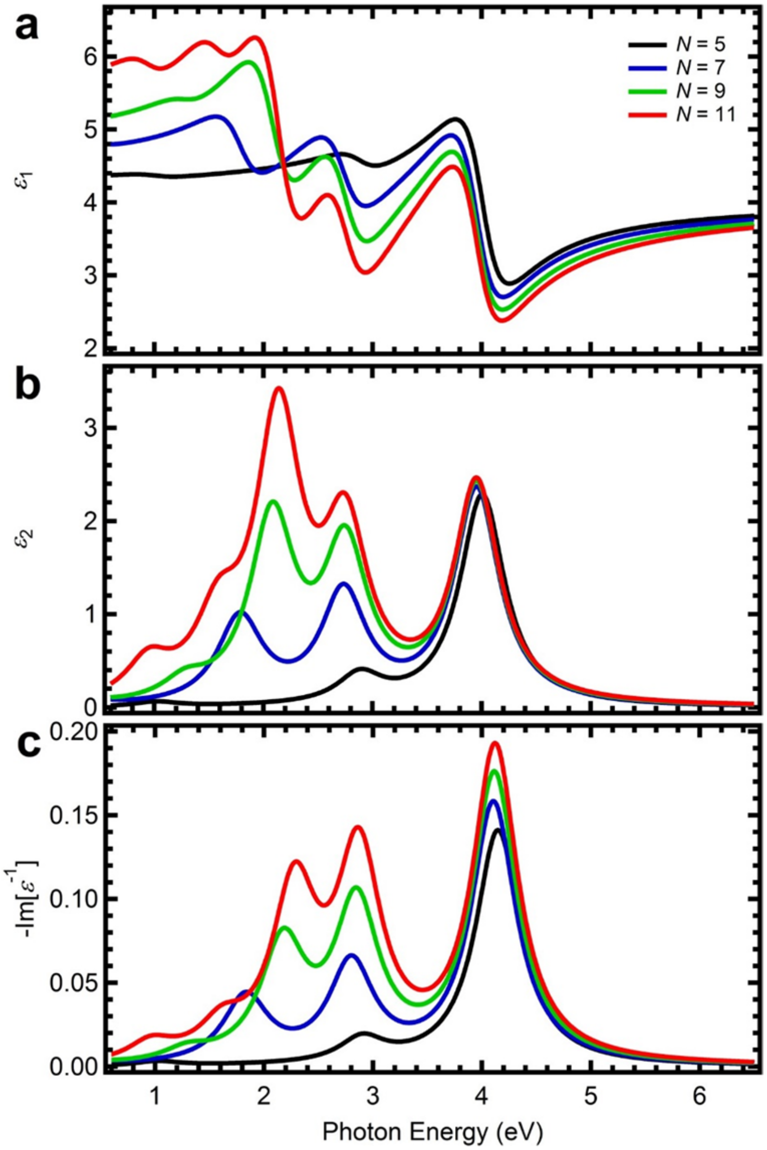

Supplementary Figure 21 | Evolution of calculated correlated plasmons as the number of oscillators in the coupled harmonic oscillator model is varied. a, Calculated real part of complex dielectric function, $\varepsilon_1(\omega)$, of correlated plasmons for $N = 5$, 7, 9, and 11, where N is the number of oscillators in the coupled oscillator model **b**, Calculated imaginary part of complex dielectric function, $\varepsilon_2(\omega)$, of correlated plasmons for $N = 5$, 7, 9, and 11. **c**, Calculated loss function, $-\text{Im}\left[\varepsilon^{-1}(\omega)\right]$, of correlated plasmons for $N = 5$, 7, 9, and 11. The legend is shown in **a**. The case of $N = 7$ gives the best resemblance to the experimental data.

Supplementary Tables

Supplementary Table 1. Properties of the second batch of pressure-dependent $Sr_{1-x}NbO_{3+\delta}$ films.

Film	Oxygen Pressure (Torr)	Thickness (nm)	Resistivity (Ωcm)	Carrier Density (cm^{-3})	Mobility ($cm^2V^{-1}s^{-1}$)
lp-SNO-2	5×10^{-6}	159	3×10^{-3}	1.3×10^{22}	2.3
mlp-SNO-2	1×10^{-5}	162	3×10^{-3}	1.3×10^{22}	2.2
mp-SNO-2	3×10^{-5}	171	4×10^{-3}	1.0×10^{22}	2.9
mhp-SNO-2	7×10^{-5}	167	6×10^{-3}	0.6×10^{22}	0.1
hp-SNO-2	1×10^{-4}	182	67	-	-
hrp-SNO-2	5×10^{-4}	180	> limit	-	-

The thicknesses of the films are obtained from spectroscopic ellipsometry analysis. The resistivity, carrier density, and mobility of the films are measured at room temperature. The hp-SNO-2 and hrp-SNO-2 films are insulating, while the rest are conducting. The resistivity of hrp-SNO-2 is beyond the measurement limit.

Supplementary Notes

Supplementary Note 1

Structurally, the $SrNbO_{3+\delta}$ family of oxides is a member of a larger group of oxides with a general formula $A_nB_nO_{3n+2}$ (Supplementary Reference 1). This structural group of oxides is also sometimes referred to as 'layered perovskites', since their crystal structure is that of perovskite 'supercells' that are layered against each other every few unit cells along one particular direction (for example [110]). Nearest-neighbor perovskite supercells have a height difference (along the [011] direction) of a half unit cell, while next-nearest-neighbor supercells have the same height. At each end of each supercell, there are half per unit cell oxygen ions that are not compensated by cations. Thus, the uncompensated oxygen ions from two neighboring supercells form an extra oxygen plane along the [101] direction, as shown in the transmission electron microscopy image in Fig. 3. The subscript n indicates the size of each perovskite supercell and thus the repetition of this pattern. In particular, for $n = 5$ we get the structure of $SrNbO_{3.4}$, while for $n = \infty$ we get the (unlayered) perovskite structure of $SrNbO_3$ with no extra oxygen planes.

Supplementary Note 2

Although the presence of the three mid-gap peaks of hp-SNO in the loss function (LF) spectrum in Fig. 1d strongly indicates that the peaks come from plasmonic excitation, there are several other interpretations such as spin-orbit coupling, phononic, excitonic, or anisotropic effects that can be used to explain these mid-gap peaks. Below, we discuss why these other interpretations are unlikely to play a role in the origin of these mid-gap peaks.

First, to check the effects of spin-orbit coupling (SOC) on the band structure and loss function of $SrNbO_3$ (which has similar stoichiometry with hp-SNO), we perform DFT calculations with that explicitly include SOC effects in its implementation. From the

calculation, we find that SOC results in the splitting of the bands with a magnitude of ~0.3-0.5 eV. This band splitting is too small compared to the energy separation among the mid-gap peaks, which has a minimum separation of more than ~1 eV. (For reference, the separation between C_1 and C_2 peaks is ~1.3 eV, while the separation between C_2 and C_3 peaks is ~1 eV.) Furthermore, we also perform random phase approximation[2] (RPA) on top of this DFT+SOC calculation to calculate the loss function (LF) of $SrNbO_{3.4}$ under the explicit inclusion of SOC effects. We find that the calculated LF spectrum only shows one single plasmon peak (similar to the calculation without SOC effects shown in Supplementary Figure 19) and is unable to mimic the three mid-gap LF peaks that we find experimentally in Fig. 1d. Thus, spin-orbit coupling is not the main reason for the existence of the three mid-gap peaks in hp-SNO.

Second, phononic peaks due to electron-phonon coupling have been observed in $SrNbO_{3.4}$, as reported by Supplementary Reference 3. However, these phononic peaks have very low energies in the range of 5-125 meV. Since the intensities of three mid-gap peaks of hp-SNO are very high and their energy positions are in the range of 1.7-4.0 eV, it is very unlikely that these mid-gap peaks come from electron-phonon coupling because their energies are simply too high for phononic interactions in this material.

Third, excitonic peaks in inorganic compounds such as strontium niobates typically have three main characteristics: they are quite sharp (with full-width half-maximum, FWHM, of typically less than 200 meV), have asymmetric shape (since exciton interactions can turn into Fano resonances), and have energy positions that are just below the bandgap (especially for Wannier bound excitons, with binding energy, measured from the exciton energy to the first interband transition energy, of typically less than 100 meV). Based on these criteria, it is clear that the three mid-gap peaks of hp-SNO at 1.7 eV, 3.0 eV, and 4.0 eV do not originate from excitonic interactions, since they are quite broad (with FWHM of ~500 meV), quite symmetric in shape, and occur quite far below the first interband transition energy of 4.6 eV.

Fourth, as we have discussed in the Method section, we found that the hp-SNO film, which possesses the three mid-gap peaks, is largely optically isotropic because its in-plane and out-of-plane dielectric functions are the same. Thus, the three mid-gap peaks of hp-SNO are unlikely to come from anisotropic effects because within our measurement and analysis limits, the hp-SNO film is optically isotropic.

Supplementary Note 3

Here, we discuss the phenomenological modellings of conventional and correlated plasmons in details. First, $SrNbO_3$ is believed to be a weakly-correlated metal[4-6], in which the on-site as well as the inter-site Coulomb repulsions between the Nb-4*d* electrons are not sufficiently strong (*i.e.*, screened) to make the system a Mott insulator[7]. In lp-SNO and mp-SNO, the appearance of the Drude peak and the conventional plasmon signatures in their complex dielectric function, $\varepsilon(\omega)=\varepsilon_1(\omega)+i\varepsilon_2(\omega)$, and loss function (LF) spectra (Figs. 1a-c and Supplementary Figure 4) indicate their metallic characteristics. On the other hand, hp-SNO is observed to be insulator-like, and its non-negative $\varepsilon_1(\omega)$ (Fig. 1a) and wide-range spectral weight transfer (Fig. 2) indicate that the Coulomb interactions among its Nb-4*d* electrons are unscreened and the electrons are strongly correlated. These electronic and optical signatures are accompanied by observations of extra oxygen planes formations every few unit cells in its atomic structure (Figs. 3c-3e), which suggests that the oxygen planes play an important role in determining the electronic properties of hp-SNO. In this regard, we argue that these oxygen planes can act as high potential barriers or walls for the originally-itinerant Nb-4*d* electrons and prevent them to hop across the planes. As a consequence, the electrons confined between two oxygen walls feel the Coulomb repulsions among them to be much stronger than they do if they are not confined. Such a situation leads the system to be much more strongly correlated and transform it into a Mott-like insulator.

Furthermore, hp-SNO is also observed to have anomalous plasmonic signatures in its complex dielectric function and LF spectra (Figs. 1a and 1d). To investigate the origin of these anomalous plasmonic signals, we use a phenomenological approach and seek for an effective model that can fit and explain the experimental optical properties of hp-SNO. Our calculation results agree in many respects with the experimental data, allowing us to propose an interpretation to explain the renormalized systems. In the following discussions, we construct two models to mimic the two observed plasmonic behaviors: (1) the conventional plasmon in conducting lp-SNO and mp-SNO and (2) the anomalous strongly-correlated plasmon in insulator-like hp-SNO.

As it is well known, bulk conventional plasmon is the quantum term for a collective oscillatory motion of charge carriers with positively charged ionic background (Fig. 6c), which mainly occurs in metals[8]. To describe this plasmon classically, it is usually sufficient to use the Drude model[8], which are valid for the energy range below the onset energy of interband transitions. Here, we present the calculations of complex dielectric function, optical conductivity, and LF spectra based on the Drude model. We start with the Newtonian equation of motion of an electron under the influence of oscillatory electric field, **E**, of the incoming light and a dissipative force proportional to its velocity,

$$m\frac{d^2\mathbf{r}}{dt^2} = -\frac{m}{\tau}\frac{d\mathbf{r}}{dt} - e\mathbf{E}(\mathbf{r},t), \quad (1)$$

where **r** denotes the displacement vector of an electron from its equilibrium position, m is the electron mass, e is the elementary charge, and τ is the lifetime in which electrons can move without experiencing a scattering process. Taking the electric field as $\mathbf{E} \cong \mathbf{E}_0 \exp(-i\omega t)$ for the long-wavelength limit, and hence $\mathbf{r}(t) \cong \mathbf{r}_0 \exp(-i\omega t)$, Supplementary Equation 1 can be rewritten as

$$-m\omega^2\mathbf{r} = i\frac{m}{\tau}\omega\mathbf{r} - e\mathbf{E}, \quad (2)$$

or,

$$\mathbf{r} = \frac{e\mathbf{E}}{\left(m\omega^2 + i\frac{m}{\tau}\omega\right)}. \tag{3}$$

The induced polarization is defined as

$$\mathbf{P} = -ne\mathbf{r} = -\frac{ne^2\mathbf{E}}{\left(m\omega^2 + i\frac{m}{\tau}\omega\right)}, \tag{4}$$

and the complex dielectric function is then derived as

$$\varepsilon(\omega) = 1 + \frac{P}{E} = 1 - \frac{ne^2}{\left(m\omega^2 + i\frac{m}{\tau}\omega\right)}, \tag{5}$$

with its real and the imaginary parts,

$$\varepsilon_1(\omega) = \mathrm{Re}\left[\varepsilon(\omega)\right] = 1 - \frac{nme^2\omega^2}{\left(m\omega^2\right)^2 + \left(\frac{m}{\tau}\omega\right)^2}, \tag{6a}$$

and

$$\varepsilon_2(\omega) = \mathrm{Im}\left[\varepsilon(\omega)\right] = \frac{ne^2\left(\frac{m}{\tau}\omega\right)}{\left(m\omega^2\right)^2 + \left(\frac{m}{\tau}\omega\right)^2}. \tag{7a}$$

Finally, adding the contribution of the core electrons of the ions in the crystal, Supplementary Equation 6a can then be corrected to be

$$\varepsilon_1(\omega) = \varepsilon_1(\infty) - \frac{nme^2\omega^2}{\left(m\omega^2\right)^2 + \left(\frac{m}{\tau}\omega\right)^2} \equiv \varepsilon_1(\infty)\left[1 - \frac{\omega_{\mathrm{p}}^2}{\omega^2 + \left(\frac{1}{\tau}\right)^2}\right], \tag{6b}$$

while Supplementary Equation 7a simplifies into

$$\varepsilon_2(\omega) = \frac{\varepsilon_1(\infty)\,\omega_p^2\left(\frac{\omega}{\tau}\right)}{\left(\omega^2\right)^2 + \left(\frac{\omega}{\tau}\right)^2}, \tag{7b}$$

with $\omega_p = \sqrt{\frac{ne^2}{\varepsilon_1(\infty)m}}$ being the conventional plasma frequency[8].

The corresponding optical conductivity and LF can then be calculated respectively as

$$\sigma_1(\omega) = \varepsilon_0 \varepsilon_2(\omega)\,\omega \tag{8}$$

and

$$\mathrm{LF} = -\mathrm{Im}\left[\varepsilon^{-1}(\omega)\right] = \frac{\varepsilon_2(\omega)}{\varepsilon_1^2(\omega) + \varepsilon_2^2(\omega)}. \tag{9}$$

Meanwhile, we refer to the plasmons observed in hp-SNO film as strongly-correlated plasmons because we believe that they result from the strong correlation effects among the Nb-4*d* electrons confined between two oxygen walls. How exactly correlation effects transform the system from originally a metal to an insulator with some peculiar plasmonic behavior is still not clear and microscopic modeling with rigorous quantum many-body treatments may be required to unravel the mechanism. At this point, such detailed microscopic study is beyond our scope, and here we simply aim to know what kind of effective system that the original system gets renormalized into as a result of correlation effects. For this purpose, we propose a hypothesis, formulate it in a classical model, and check if the model results in calculated $\varepsilon(\omega)$ and LF spectra that can closely resemble the experimental data.

We start by considering the three peaks at ~1.7 eV, ~3.0 eV, and ~4.0 eV of the LF spectra (Fig. 1d). Since these peaks appear in the LF spectra, they should correspond to plasmonic excitations in the limit where the transferred momentum approaches zero[9], which is the case for sub-X-ray photon excitations. However, they cannot be conventional plasmons

since their energies do not coincide with the zeroes of $\varepsilon_1(\omega)$, as $\varepsilon_1(\omega)$ has no zeroes in that energy region. In this case, we hypothesize that these plasmonic signatures originate from oscillatory motions of the confined electrons between two oxygen walls. As the originally-itinerant Nb-*4d* electrons are trapped in the confined region, increased Coulomb repulsions among them induce localization of the charges at Nb sites, which increases their correlations. This is supported by density functional theory (DFT) calculations results in Fig. 6b that shows that the presence of oxygen planes in $SrNbO_{3.4}$, which shares similar extra oxygen plane structure with hp-SNO, can indeed induce a confinement effect to Nb-4*d* electrons.

Then, we next hypothesize that these correlation effects transform the system in such a way that the charged quasi-particles localized at each Nb site have different characters compared to those of the original electrons, *i.e.*, their effective masses and charges may be different. Further, the quasi-particle at each site feels the effective Coulomb interactions from its neighbors that puts it into a potential well, which for simplicity we approximate as a harmonic oscillator potential. From this, we propose that the effective system manifests itself as a chain of quasi-particles with a spring-like force that couples every two neighboring quasi-particles, while each of the two quasi-particles near the oxygen walls is attached to the wall through a spring-like force as well. As the relevant length of the chain is still not known, we shall take the chain length as a variable that we vary in our calculations, and later check which length gives the results that best resemble the experimental data. Thus, the strongly-correlated plasmons are modelled as a system of coupled one-dimensional N-body harmonic oscillator with N quasi particles and N+1 springs. The effective model is illustrated in Fig. 6d and Supplementary Figure 20.

Corresponding to the model described above, we formulate a set of N Newtonian equation of motions for N coupled quasi-particles as follows,

$$m_1 \frac{d^2u_1}{dt^2} = -k_1u_1 - k_2\left(u_1 - u_2\right) - \frac{m_1}{\tau}\frac{du_1}{dt} + q_1E\ ,$$

$$m_2 \frac{d^2u_2}{dt^2} = -k_2\left(u_2 - u_1\right) - k_3\left(u_3 - u_2\right) - \frac{m_2}{\tau}\frac{du_2}{dt} + q_2E\ ,$$

$$\cdot$$

$$\cdot$$

$$\cdot$$

$$m_{N-1} \frac{d^2u_{N-1}}{dt^2} = -k_{N-1}\left(u_{N-1} - u_{N-2}\right) - k_N\left(u_N - u_{N-1}\right) - \frac{m_{N-1}}{\tau}\frac{du_{N-1}}{dt} + q_{N-1}E\ ,$$

$$m_N \frac{d^2u_N}{dt^2} = -k_N\left(u_N - u_{N-1}\right) - k_{N+1}\left(-u_N\right) - \frac{m_N}{\tau}\frac{du_N}{dt} + q_NE\ , \tag{10}$$

where m_i, u_i, and q_i is the mass, displacement, and charge of the i-th quasi-particle, respectively, while k_i is the spring constant of the i-th spring. Taking $E \cong E_0 \exp\left(-i\omega t\right)$ for the long-wavelength limit, and $u\left(t\right) = u_0 \exp\left(-i\omega t\right)$ as before, the above set of equations can then be re-written in a matrix form as

$$\mathbf{M}\begin{pmatrix} u_1 \\ u_2 \\ u_3 \\ \vdots \\ u_N \end{pmatrix} = \begin{pmatrix} q_1E \\ q_2E \\ q_3E \\ \vdots \\ q_NE \end{pmatrix}, \tag{11}$$

where $\mathbf{M}$ is an $N \times N$ symmetric matrix whose non-zero diagonal and off-diagonal elements are of the form

$$M_{i,i} = -m_i\omega^2 + k_i + k_{i+1} - i\frac{m_i}{\tau}\omega \tag{12}$$

and

$$M_{i,i+1} = M_{i+1,i} = -k_{i+1}\ , \tag{13}$$

respectively. The quasi particle displacements can be extracted as

$$\begin{pmatrix} u_1 \\ u_2 \\ u_3 \\ \vdots \\ u_N \end{pmatrix} = \mathbf{M}^{-1} \begin{pmatrix} q_1 E \\ q_2 E \\ q_3 E \\ \vdots \\ q_N E \end{pmatrix}, \tag{14}$$

and the corresponding polarization can be calculated as

$$P = \sum_{i=1}^{N} n_i q_i u_i \ , \tag{15}$$

with $n_i = \frac{1}{V_{\text{cell}}}$ being the local density of the quasi particle at the corresponding unit cell. The volume of film unit cell, V_{cell}, is calculated from the lattice constants obtained from XRD (Supplementary Figure 1) using cubic perovskite structure. Finally, the complex dielectric function is defined as

$$\varepsilon(\omega) = \varepsilon_1(\infty) + \frac{P}{E}, \tag{16}$$

and the optical properties $\varepsilon_1(\omega) = \text{Re}\left[\varepsilon(\omega)\right]$, $\varepsilon_2(\omega) = \text{Im}\left[\varepsilon(\omega)\right]$, $\sigma_1(\omega) = \varepsilon_0 \varepsilon_2(\omega)\omega$, and $\text{LF} = -\text{Im}\left[\varepsilon^{-1}(\omega)\right]$ are computed from $\varepsilon(\omega)$ accordingly.

The calculated $\varepsilon(\omega)$ and LF spectra of the conventional plasmons are shown in Figs. 6e,f. For the calculations, we take the following parameters: $m = m_e$, $n = \frac{1}{V_{\text{cell}}}$, ω_{p} = 2 eV, $\varepsilon(\infty) = \frac{ne^2}{m\omega_{\text{p}}^2}$, and $\frac{1}{\tau}$ = 0.5 eV, where m_e is the elementary electron mass. With this choice of parameters, our calculation results are in a very good agreement with the experimental data for the energy range up to 4 eV, *i.e.*, below the onset energy of the interband transitions, confirming that the lp-SNO and mp-SNO are metals with conventional plasmonic characteristics. Furthermore, as it is well understood, the LF of the conventional plasmons

reveals a plasmonic peak at the position coinciding with that of the zero of $\varepsilon_1(\omega)$, while $\varepsilon_2(\omega)$ does not have any peak structure for $\omega > 0$.

For the strongly-correlated plasmons, the first step is to find what N value yields the results that resemble the experimental data the best. In analogy with classical mechanics, an N-oscillator system has N natural oscillation modes. Since the number of observed plasmonic peaks in Fig. 1d is three, this gives an indication that N should be an odd number. We start with the case of $N = 3$. A 3-body oscillator have three intrinsic oscillation modes, but there is one mode that has zero polarization. This zero-polarization mode cannot show up in $\varepsilon(\omega)$ spectra, leaving only two non-zero peaks and inconsistent with the three peaks observed in Fig. 1d. Thus, the chain orientation corresponding to $N = 3$ is not consistent with experimental observations.

Next, for $N = 5$ the 5-body oscillator has five eigenmodes, but two of them have zero polarization, leaving three non-zero peaks surviving, which is what the experimental results require. At this point, besides getting the correct number of peaks and the correct peak positions, we also need to make sure that the peak intensity indeed increases as energy increases, as observed in Fig. 1d. If we assume all the charge of the quasi-particles to be the same as that of a bare electron, the calculation results in a peak intensity trend that decreases as energy increases, which is the opposite trend compared to what observed experimentally. This happens because the slower vibration mode corresponds to higher polarization, which gives higher peak intensity as energy increases. To improve this, we find that the increasing intensity trend can only be achieved if the charges are assumed to be distributed with alternating signs, *i.e.*, $q_1 = -e$, $q_2 = +e$, $q_3 = -e$, and so on. This is a surprising phenomenological observation as it might suggest an interpretation that the originally itinerant electrons have transformed into quasi-electrons and quasi-holes distributed

alternatingly along the chain. This is consistent with previous report of possible charge density wave existence in $SrNbO_{3.4}$ (Supplementary Reference 10).

In order to tune the peak positions, we first set an energy scale of about the lowest energy of the three plasmonic peaks, *i.e.*, ω_0 = 2.0 eV. We also set a scale for the effective mass values to be $m^* = Zm_e$, and a scale for the spring constant values to be $k_0 = m^*\omega_0^2$. Here, Z is the mass renormalization factor that we initially set to be equal to 1 and adjust it as necessary. As initial guesses, we set $m_1 = m_2 = \ldots = m_N = m^*$ and $k_1 = k_2 = \ldots = k_{N+1} = k_0$. Fine-tuning of the peak positions is done by adjusting individually each $k_i(m_i)$ slightly away from $k_0(m^*)$. The Z value is also fine-tuned so that the magnitudes of the calculated $\varepsilon_1(\omega)$ and $\varepsilon_2(\omega)$ match closely with experimental data. Interestingly, to obtained the correct magnitudes of $\varepsilon_1(\omega)$ and $\varepsilon_2(\omega)$, the Z value needs to be set to $Z \approx 25$, which suggests that the quasi-particle masses are heavily renormalized due to strong correlations. The calculation results are presented in Supplementary Figure 21, which shows that for $N = 5$ the peak positions and the increasing intensity trend are in a rough agreement with experimental results (Fig. 1d). However, the calculated relative intensities among the peaks are not very satisfactory because the first and the second peaks are too low compared to the third peak.

To improve the calculation results, the calculation for the case of $N = 7$ is next performed with the same approach as discussed above. Interestingly, not only does it give three appearing peaks (Figs. 6e,g and Supplementary Figure 21), with only an additional negligibly small peak close to 0 eV, but also it provides a peak intensity profile that much better resembles the experimental results (Fig. 1d). For this particular case, we set the parameter values as follows: ω_0 = 2.0 eV, Z = 25, $\varepsilon_1(\infty)$ = 4.0, $\frac{1}{\tau}$ = 0.5 eV, $m_1 = m_2 = \ldots = m_N = Zm_e$, $k_1 = 0.4k_0$, $k_2 = 0.5k_0$, $k_3 = 1.0k_0$, $k_4 = 1.1k_0$, and the rest of the

spring constants follow a mirror symmetry, *i.e.*, $k_8 = k_1$, $k_7 = k_2$, $k_6 = k_3$, and $k_5 = k_4$. The calculations are also performed for $N = 9$ and $N = 11$, although Supplementary Figure 21 shows that these two cases result in too many peaks, inconsistent with experimental results. Thus, the phenomenological modelling shows that the strongly-correlated plasmons in hp-SNO can be explained using a coupled seven-oscillator model.

Supplementary Note 4

From Supplementary Figure 14g, it can be seen that the $\varepsilon_2(\omega)$ and LF spectra of the insulating hp-SNO-2 film (deposited under the same high oxygen pressure as the main-batch hp-SNO) also show the distinct signatures of multiple correlated plasmon peaks. Interestingly, the number of correlated plasmon peaks in hp-SNO-2 (five) is higher than in hp-SNO (three), with two new correlated plasmon peaks appearing in the lower energy region below ~1.4 eV that were absent in hp-SNO. Furthermore, the energies of the three highest correlated plasmon peaks in hp-SNO-2 (~2.4, ~3.5, and ~4.1 eV) are slightly higher than the three correlated plasmon peaks of hp-SNO (~1.7, ~3.0, and ~4.0 eV). These indicate that the second-batch hp-SNO-2 film may have a slightly higher electronic correlation than hp-SNO, as corroborated by its resistivity that is ~11 times higher than that of hp-SNO (Supplementary Table 1). This is perhaps due to the various (not fully controllable) dynamical processes that can affect film growth in pulsed-laser depositions. From Supplementary Figure 21, it can be seen that when the number of oscillators between the oxygen wall is increased above seven, more correlated plasmon peaks start to appear especially at the lower energy region. Thus, the appearance of extra correlated plasmon peaks in hp-SNO-2 (and hrp-SNO-2, see below) can be explained by these higher-order modes in our coupled oscillator model. Nevertheless, the appearance of distinct correlated plasmon peaks in hp-SNO-2 indicates that the correlated plasmons are reproducible and not only appear in one particular sample.

In higher-pressure hrp-SNO-2, the correlated plasmon peaks are even more prominent (particularly the two lowest-energy peaks) and slightly blue-shifted. On the other hand, when free-charge density is increased in mhp-SNO-2 and the film becomes conducting, these correlated plasmons start to diminish (only two correlated plasmons at ~2.3 and ~3.9 eV are observed in mhp-SNO-2) and ultimately vanish in lp-SNO-2, consistent with correlated plasmon behaviour of the main-batch films shown in Fig. 1.

The behaviors of conventional plasmon and excitonic peaks in these second-batch films are also very similar with those of the main-batch films. From Supplementary Figure 14b, it can be seen that lp-SNO-2 film has the highest conventional bulk plasmon energy (among the second-batch films) at ~1.8 eV. This conventional plasmon gradually red-shifts as the free-charge density decreases (down to ~1.2 eV in mhp-SNO-2), and ultimately vanishes in insulating hp-SNO-2 and hrp-SNO-2 films. This is consistent with the conventional plasmon behaviour of the main-batch films in Fig. 1 and in contrast with the correlated plasmon behaviour described above. Furthermore, the insulating hp-SNO-2 and hrp-SNO-2 films also show a distinct excitonic peak at ~4.5 eV (Supplementary Figures 14g,h), and this excitonic peak gradually diminish and ultimately vanish as free-charge density increases, similar to the excitonic peak behaviour of the main-batch films shown in Fig. 1.

Optical conductivity and W analyses of these second-batch films (Supplementary Figure 16) also show that their spectral weight transfer behaviour is very similar to that of the main-batch films. As W_1 (Drude region) decreases from lp-SNO-2 to mhp-SNO-2, W_3 (first interband transition region) increases due to the increased availability of unoccupied Nb-4d states. However, when MIT happens between mhp-SNO-2 and hp-SNO-2, both W_1 and W_3 anomalously decrease, leading to an overall decrease of W below 6.3 eV (W_{tot}). Again, according to the f-sum rule[9,11,12], this decrease has to be compensated by an equivalent increase above 6.3 eV, implying anomalous wide-range spectral weight transfers on the onset

of the MIT, which is a direct evidence of strong correlations[7,9,12-17] in hp-SNO-2. The W_3 and W_{tot} continue to decrease in hrp-SNO-2, implying even larger anomalous spectral weight transfers and thus stronger electronic correlations than in hp-SNO-2, which enhances the correlated plasmons as shown in Supplementary Figure 14h. Thus, the pressure-dependent MIT is also reproducible, and that the films deposited under higher oxygen pressures are consistently shown to have the signature of strong electronic correlations.

Supplementary Note 5

The theoretical LF spectra of $SrNbO_3$ and $SrNbO_3$ shown in Supplementary Figure 19 are calculated using density functional theory (DFT) with random phase approximation (RPA) method[2], which, by itself, cannot include the effects of electronic correlations properly. Supplementary Figure 19a shows that according to this calculation, the LF of $SrNbO_3$ should have a conventional plasmon peak and a structure above the first interband gap of 4.6 eV. This is similar to the LF of lp-SNO, albeit with apparent differences in the conventional plasmon peak energy and intensity which can be explained by the tendency of the ground state from DFT calculations to underestimate the bandgap and the density of metallic electrons[18]. This ability of DFT+RPA to qualitatively mimic the LF of lp-SNO is not surprising because lp-SNO is a metal with little to no correlation effects.

On the other hand, it can be seen in Supplementary Figure 19b that without correlations, DFT+RPA calculation cannot qualitatively mimic the LF of hp-SNO, particularly the three mid-gap peaks observed experimentally in hp-SNO. Note that in RPA, plasmons are treated as superpositions of electron-hole pairs of bare electrons without spectral weight transfers. This time, the differences cannot be attributed to DFT bandgap underestimation anymore because the experimental LF spectrum contains three plasmonic peaks that cannot be explained simply by Drude model. Based on our analysis, this is where

correlations play an important role, and thus it is not surprising that DFT+RPA cannot predict these three peaks because it is unable to properly include correlation effects to begin with.

The loss function of $SrNbO_{3.4}$ is also calculated using RPA with explicit spin-orbit coupling (SOC) implementation. To accelerate calculations, $SrNbO_{3.4}$ is modeled by standard cubic perovskite structure with 5 atoms and primitive cell with 27 atoms respectively. The *k*-point meshes are 8×6×2 to sample the Brillouin zone. Number of bands are increased which doubled the default values which up to 256 bands. Symmetries are switched off to compare energies for different orientations and to determine the magnetic anisotropies. Initial magnetic moment for all Nb atoms are parallel to z-direction (out of plane) with 1.0 μ_B. We find that the calculated loss function of $SrNb_{3.4}$ after explicit inclusion of SOC effects only results in one plasmon frequency (instead of three), similar with the calculation result without SOC implementation.

Supplementary Note 6

The Sr/Nb cationic ratio of the films is determined using Rutherford backscattering spectrometry to be 0.94, which means that the films have 6% Sr vacancies in their composition (see Methods). We do not detect the presence of other cationic vacancies within our measurement resolution. As for the O concentration, it remains very challenging to quantify precisely the O content in films, particularly in oxide films on oxide substrates such as the lp-SNO, mp-SNO, and hp-SNO films that are deposited on $LaAlO_3$ substrates. Nevertheless, as discussed above and in Supplementary Reference 1, $SrNbO_{3+\delta}$ with different oxygen content (*i.e.*, different δ, with $\delta \geq 0$) has different extra oxygen plane spacing and repetition pattern. Thus, in this case the oxygen content of the lp-SNO, mp-SNO, and hp-SNO films can be estimated by considering the local oxygen defect microstructures of the films. From the transmission electron microscopy (TEM) image in Fig. 3e, it can be seen that

the extra oxygen planes structure of hp-SNO matches with that of bulk $SrNbO_{3.4}$, which means that hp-SNO should have an oxygen content close to that of $SrNbO_{3.4}$. On the other hand, Fig. 3a shows that lp-SNO has almost no extra oxygen planes, which means that it should have an oxygen content close to that of $SrNbO_3$. Meanwhile, since mp-SNO is deposited under an oxygen pressure between those of lp-SNO and hp-SNO, it should also have an oxygen content between those of lp-SNO and hp-SNO. The density of the oxygen plane has also been measured using selected-area electron diffraction (SAED) mode of TEM (Supplementary Figure 8), where it is shown that lp-SNO has almost no extra oxygen planes while the density of extra oxygen planes in hp-SNO is four times higher than in mp-SNO. However, unfortunately it is not straightforward to convert these oxygen plane density values into actual oxygen concentration in the films.

Supplementary Note 7

Spectroscopic ellipsometry analysis (Supplementary Figure 5) and XRD results (Supplementary Figure 1) suggest that the hp-SNO film is optically isotropic. Meanwhile, previous studies have reported that bulk $SrNbO_{3.4}$, which shares similar extra oxygen plane structure with hp-SNO, is highly anisotropic along the direction of the extra oxygen planes[10,19-21]. This difference can be explained as follows. In those previous studies, the extra oxygen planes were oriented only along the [101] direction, providing a distinct direction for unambiguous measurements of the anisotropy of bulk $SrNbO_{3.4}$. On the other hand, in this study the global atomic arrangement of the extra oxygen planes is such that the planes are oriented along both [101] and [-101] directions (Fig. 3c), which are perpendicular to each other. This causes twin domains to be formed in the film structure based on the direction of the oxygen planes: on some domains, the oxygen planes are along the [101] direction, while on others they are along the [-101] direction. From the scale bar in Fig. 3c (8 nm), it can be

estimated that the width of one of these 'orientation domains' is ~24 nm. Since the beam spot size used in the spectroscopic ellipsometry measurements is ~1-3 mm, the contributions from these domains become macroscopically averaged over a large area, which leads to the apparent isotropy of hp-SNO film in the optical data.

Supplementary Note 8

In addition to $SrNbO_3$ and $SrNbO_{3.4}$ (Figs. 6a,b), we also perform density functional theory (DFT) calculations on $SrNbO_{3.33}$ and $SrNbO_{3.5}$ (Supplementary Figure 18). From these calculations, the effect of different oxygen concentration in $SrNbO_{3+\delta}$ is to change the spacing (and thus density) of the extra oxygen planes. Thus, if the extra oxygen planed density is changed, we expect the electronic confinement between the oxygen planes to also change. Again, it should be noted that DFT calculations are not able to explain the spectral weight transfer observed in Fig. 2, as this requires electronic correlations which are not included in the current calculations.

If the oxygen concentration is decreased to $SrNbO_{3.33}$ (Supplementary Figure 18a), the extra oxygen spacing would increase to 6 unit cells (for $SrNbO_{3.4}$ the spacing is 5 unit cells). Because of this, we expect a decrease in the electron confinement and, by extension, a decrease in the electronic correlation. This decrease in the electronic correlation would diminish the correlated plasmons, as seen in the $\varepsilon(\omega)$ of mp-SNO (which is estimated to have a lower oxygen concentration compared to hp-SNO) where there is only one small correlated plasmon peak at ~3.5 eV (B_2' peak in Fig. 1c). On the other hand, if the oxygen concentration is increased to $SrNbO_{3.5}$ (Supplementary Figure 18b), the extra oxygen spacing would decrease to 4 unit cells. However, this increase of extra oxygens is enough to deplete the confined electrons, and as a result $SrNbO_{3.5}$ is found to be an (ferroelectric) insulator[22,23].

Another remark from the DFT calculations is that we also find different orbital configurations for each cases, which may be related to the different types of plasmons that are excitable in different films: conventional plasmons in $SrNbO_3$, correlated plasmons in $SrNbO_{3.4}$ and a mixture of conventional and correlated plasmon in $SrNbO_{3.33}$. Since DFT calculations cannot clarify the existence of correlated plasmons, at this stage it remains to be seen how to quantitatively link between these different orbital configurations with various types of plasmons.

Supplementary References

1. Lichtenberg, F., Herrnberger, A., Wiedenmann, K. & Mannhart, J. Synthesis of perovskite-related layered $A_nB_nO_{3n+2} = ABO_x$ type niobates and titanates and study of their structural, electric and magnetic properties. *Prog. Solid State Chem.* **29**, 1-70 (2001).
2. Onida, G., Reining, L. & Rubio, A. Electronic excitations: density-functional versus many-body Green's-function approaches. *Rev. Mod. Phys.* **74**, 601-659 (2002).
3. Kuntscher, C. A. *et al.* Extremely small energy gap in the quasi-one-dimensional conducting chain compound $SrNbO_{3.4}$. *Phys. Rev. Lett.* **89**, 236403 (2002).
4. Isawa, K., Sugiyama, J., Matsuura, K., Nozaki, A. & Yamauchi, H. Synthesis and transport properties of Sr_xNbO_3 ($0.75 \leq x \leq 0.90$). *Phys. Rev. B* **47**, 2849-2853 (1993).
5. Xu, X., Randorn, C., Efstathiou, P. & Irvine, J. T. S. A red metallic oxide photocatalyst. *Nat. Mater.* **11**, 595-598 (2012).
6. Chen, C. *et al.* Atomic and electronic structure of the $SrNbO_3/SrNbO_{3.4}$ interface. *Appl. Phys. Lett.* **105**, 221602 (2014).
7. Imada, M., Fujimori, A. & Tokura, Y. Metal-insulator transitions. *Rev. Mod. Phys.* **70**, 1039-1263 (1998).
8. Maier, S. A. *Plasmonics: Fundamentals and Applications* (Springer, 2007).

9. Van der Marel, D. in *Strongly Correlated Systems* Vol. 180 *Springer Series in Solid-State Sciences* (eds. Adolfo Avella & Ferdinando Mancini) Ch. 9, 269-296 (Springer Berlin Heidelberg, 2015).

10. Weber, J. E. *et al.* NMR, EPR, and bulk susceptibility measurements of one-dimensional $SrNbO_{3.4}$. *Phys. Rev. B* **64**, 235414 (2001).

11. Kubo, R. Statistical-mechanical theory of irreversible processes. I. General theory and simple applications to magnetic and conduction problems. *J. Phys. Soc. Jpn.* **12**, 570-586 (1957).

12. Basov, D. N., Averitt, R. D., van der Marel, D., Dressel, M. & Haule, K. Electrodynamics of correlated electron materials. *Rev. Mod. Phys.* **83**, 471-541 (2011).

13. Eskes, H., Meinders, M. B. J. & Sawatzky, G. A. Anomalous transfer of spectral weight in doped strongly correlated systems. *Phys. Rev. Lett.* **67**, 1035-1038 (1991).

14. Meinders, M. B. J., Eskes, H. & Sawatzky, G. A. Spectral-weight transfer: Breakdown of low-energy-scale sum rules in correlated systems. *Phys. Rev. B* **48**, 3916-3926 (1993).

15. Basov, D. N. & Timusk, T. Electrodynamics of high-T_c superconductors. *Rev. Mod. Phys.* **77**, 721-779 (2005).

16. Qazilbash, M. M. *et al*. Electrodynamics of the vanadium oxides VO_2 and V_2O_3. *Phys. Rev. B* **77**, 115121 (2008).

17. Rusydi, A. *et al.* Metal-insulator transition in manganites: Changes in optical conductivity up to 22 eV. *Phys. Rev. B* **78**, 125110 (2008).

18. Sponza, L., Véniard, V., Sottile, F., Giorgetti, C. & Reining, L. Role of localized electrons in electron-hole interaction: The case of $SrTiO_3$. *Phys. Rev. B* **87**, 235102 (2013).

19. Kuntscher, C. A. *et al.* Extremely small energy gap in the quasi-one-dimensional conducting chain compound $SrNbO_{3.4}$. *Phys. Rev. Lett.* **89**, 236403 (2002).

20. Sakai, A., Kanno, T., Takahashi, K., Yamada, Y. & Adachi, H. Large anisotropic

thermoelectricity in perovskite related layered structure: $Sr_nNb_nO_{3n+2}$ (n = 4,5). *J. Appl. Phys.* **108**, 103706 (2010).

21. Kobayashi, W. *et al.* Anisotropic thermoelectric properties associated with dimensional crossover in quasi-one-dimensional $SrNbO_{3.4+d}$ (d ~ 0.03). *Phys. Rev. B* **84**, 085118 (2011).

22. Nanamatsu, S., Kimura, M. & Kawamura, T. Crystallographic and dielectric properties of ferroelectric $A_2B_2O_7$ (A = Sr, B = Ta, Nb) crystals and their solid solutions. *J. Phys. Soc. Jpn.* **38**, 817-824 (1975).

23. Akishige, Y., Kobayashi, M., Ohi, K. & Sawaguchi, E. Dielectric and ferroelectric properties in the low temperature phase of strontium niobate. *J. Phys. Soc. Jpn.* **55**, 2270-2277 (1986).
